# Unraveling DDIT4 in the VDR-mTOR pathway: a novel target for drug discovery in diabetic kidney disease

**DOI:** 10.3389/fphar.2024.1344113

**Published:** 2024-03-19

**Authors:** Hai-tao Lu, Yuan-yuan Jiao, Tian-yu Yu, Jing-xuan Shi, Jing-wei Tian, Gu-ming Zou, Lin Liu, Li Zhuo

**Affiliations:** ^1^ Department of Nephrology, China-Japan Friendship Hospital, Beijing, China; ^2^ Department of Nephrology, Fuwai Hospital, Chinese Academy of Medical Science, Beijing, China; ^3^ Institute of Clinical Medical Sciences, China-Japan Friendship Hospital, Beijing, China; ^4^ Department of Nephrology, Beijing Sixth Hospital, Beijing, China; ^5^ Capital Medical University China-Japan Friendship School of Clinical Medicine, Beijing, China

**Keywords:** diabetic kidney disease (DKD), DNA damage inducing transcription factor 4 (DDIT4), vitamin D receptor, mammalian target of rapamycin (mTOR) signal pathway, autophagy, oxidative stress

## Abstract

**Introduction:**

Diabetic kidney disease (DKD) necessitates innovative therapeutic strategies. This study delves into the role of DNA damage-inducing transcription factor 4 (DDIT4) within the VDR-mTOR pathway, aiming to identify a novel target for DKD drug discovery.

**Methods:**

Transcriptome data from the Gene Expression Omnibus Database were analyzed to assess the expression of mTOR and VDR expression in human renal tissues. Clinical samples from DKD patients and minimal change disease (MCD) controls were examined, and a DKD animal model using 20-week-old db/db mice was established. DDIT4 plasmid transfection was employed to modulate the VDR-mTOR pathway, with its components evaluated using immunohistochemistry, real-time quantitative PCR (qRT-PCR), Western blotting, and enzyme-linked immunosorbent assay (ELISA).

**Results:**

Changes in the expression of the VDR-mTOR pathway were observed in both DKD patients and the animal model. Overexpression of DDIT4 increased VDR expression and decreased levels of mTOR, p70s6k, and 4E-BP1. Furthermore, DDIT4 treatment regulated autophagy by upregulating LC3I expression and downregulating LC3II expression. Notably, DDIT4 alleviated oxidative stress by reducing the levels of lipid peroxidation product MDA, while simultaneously increasing the levels of superoxide dismutase (SOD) and glutathione (GSH), underscoring the role of DDIT4 in the pathological process of DKD and its potential as a therapeutic target.

**Conclusion:**

Unraveling DDIT4’s involvement in the VDR-mTOR pathway provides insights for innovative DKD drug discovery, emphasizing its potential as a therapeutic target for future interventions.

## 1 Introduction

The global diabetes incidence is rising sharply, becoming a major public health challenge. Projections estimate around 537 million adults with diabetes worldwide by 2021, with China leading at an expected 140.9 million cases (Edition, 2022). This upward trend is expected to continue, with an estimated rise to 174.4 million diabetic patients in China by 2045 (Sun et al., 2022). Diabetic complications vary widely, with Diabetic Kidney Disease (DKD) being a key microvascular complication marked by edema, proteinuria, and declining renal function, which may lead to renal failure and end-stage renal disease (ESRD) (Afkarian et al., 2016; Afkarian et al., 2016). Existing therapeutic strategies, encompassing blood sugar control, lipid regulation, and blood pressure management, face limitations in effectively slowing DKD progression (Alicic and Nicholas, 2022). Microalbuminuria, a key indicator of DKD progression and an independent predictor of renal function deterioration, underscores the importance of controlling albuminuria as a crucial strategy to impede DKD advancement (, 2020), (McGrath and Edi, 2019; Barrera-Chimal and Jaisser, 2020).

Recent studies highlight vitamin D and its receptor (VDR) agonists, like calcitriol and paricalcitol, as potentially effective in reducing proteinuria in DKD patients (Song et al., 2021). Vitamin D, a fat-soluble steroid derivative, undergoes metabolism into its active form, 1.25(OH)_2_D_3_ (Abasheva et al., 2022). Emerging evidence suggests that 1.25(OH)_2_D_3_ may impede the mammalian target of rapamycin (mTOR) signaling pathway by upregulating DNA damage-inducible transcript 4 (DDIT4) (Yang et al., 2015; Wang et al., 2016), presenting potential avenues for halting DKD progression. Moreover, oxidative stress elevation and diminished antioxidant capacity contribute to diabetic renal injury (Li et al., 2021; Yang and Yang, 2022). Selective autophagy mediated by nuclear receptor coactivator 4 (NCOA4), indicated by ferritin degradation, demonstrates an association with DKD (Fuhrmann et al., 2020; Stockwell, 2022).

DDIT4, also known as regulated in development and DNA damage response 1 (REDD1) or Di92, located on human chromosome 10, responds to various stimuli, playing pivotal roles in hypoxia, cell damage, and diverse stress conditions (Wang et al., 2019; Ding et al., 2021; Miller et al., 2021). This highly conserved gene exhibits increased expression in response to hypoxia, oxidative stress, DNA damage, and other stressors, influencing processes like autophagy, apoptosis, and energy stress (Wang et al., 2019; Ding et al., 2021; Miller et al., 2021). Our preliminary research implies that modulating DDIT4 could impact mTOR signaling (Chen et al., 2018a), suggesting its potential importance in VDR-related pathways and autophagy regulation. This study aims to elucidate DDIT4 expression changes and VDR-related pathway activation in DKD patients and experimental mouse models while exploring DDIT4’s role in DKD treatment and its impact on autophagy. The findings offer potential novel therapeutic strategies for managing DKD.

## 2 Materials and methods

### 2.1 Experimental design

We obtained mTOR and VDR transcriptional expression data for human renal tissue from the Gene Expression Omnibus Database (GSE142025). Clinical samples from DKD patients and minimal change disease (MCD) patients served as controls. Additionally, 20-week-old male SPF db/db mice and their littermates were used as an animal model of DKD. The study received approval from the Human Ethics Review Committee of the China-Japan Friendship Hospital (Approval Number: 2018-45-K34). All volunteers provided their informed consent and signed the necessary documents. All animal experiments complied with ARRIVE guidelines and the U.K. Animals (Scientific Procedures) Act of 1986.

The study followed these steps:

1) Transcriptome data analysis: Gene set enrichment analysis (GSEA) was performed to analyze mTOR and VDR in the transcriptional dataset.
2) Histological analysis: Kidney tissue samples from both human patients and mouse models were subjected to Periodic acid Schiff (PAS) staining to assess tissue pathology and glomerulosclerosis.
3) Immunohistochemical (IHC) staining: Molecular expression of mTOR, 4E-BP1, p70s6k and VDR was examined in clinical and mouse kidney tissues.
4) Cell Culture: MPC5 and SV40-MES-13 cell lines were cultured under high-glucose conditions to create a diabetic model, followed by cell transfection with the DDIT4 plasmid.
5) Autophagy analysis: Detection of autophagy marker proteins.
6) Molecular analysis: qRT-PCR and Western blotting were conducted to measure gene and protein expression levels.
7) Oxidative stress assessment: ELISA was used to measure the activities of MDA, SOD, and GSH in cells.

### 2.2 Transcriptome data of mTOR and VDR in human renal tissue

All transcriptional expression levels of mTOR and VDR in renal tissue from DKD and normal patients were obtained from the Gene Expression Omnibus Database (GSE142025) at the following link: https://www.ncbi.nlm.nih.gov/geo/.

### 2.3 Periodic acid schiff (PAS) staining

Mouse kidney tissue was fixed in 4% paraformaldehyde, embedded, and sectioned at 4 μm thickness. The slices were dewaxed with xylene, hydrated with a gradient of ethanol, stained with alcian blue dye for 10–20 min, oxidized with 1% periodate for 5 min, stained with Schiff dye for 10–20 min, counterstained with hematoxylin dye for 1–2 min, differentiated with a 1% hydrochloric acid ethanol solution for 2–3 s, turned blue with ammonia for 3min, dehydrated with gradient ethanol, and made transparent with dimethylbenzene. After sealing with neutral gum, slices were examined and photographed microscopically. PAS scoring criteria are as follows: 0 point, no sclerosis; 1 point, ≤10% glomerulosclerosis; 2 points, 10%–25% glomerulosclerosis; 3 points, 25%–50% glomerulosclerosis; 4 points, >50% glomerulosclerosis.

### 2.4 Immunohistochemical (IHC) staining

Clinical kidney samples and mouse kidney tissues were fixed with 4% paraformaldehyde, and after paraffin embedding, continuous sectioning was performed at a thickness of 4 μM. The slices were rehydrated with gradient ethanol solution, repaired with citrate antigen repair solution at 95°C for 15min, inactivated with H_2_O_2_ at room temperature for 10min, permeabilized with 0.1% Triton X-100 at room temperature for 10min, and blocked with a 5% BSA sealing solution at 37°C for 1 h. Subsequently, we applied primary antibodies (mTOR, 4E-BP1, p70s6k, and VDR) dropwise and incubated them at 37°C for 2 h. The corresponding secondary antibodies were then added dropwise and incubated at room temperature for 20 min, The DAB chromogenic agent was then added for 5–10 min, and the reaction was terminated using hydrogen peroxide and hematoxylin was used for nuclear staining for 1 min. Gradient ethanol dehydration, xylene transparent, drop neutral gum film, the stained slices were observed under a microscope and photographed.

### 2.5 Cell culture

MPC5 cells, cultured in DMEM high-glucose medium, were treated with 10% FBS, 1% penicillin-streptomycin, and 10U/mL γ-interferon. When the cells covered 70%–80% of the bottom area of the bottle, cells were collected to remove γ- Interferon. MPC5 cells were further cultured and induced to differentiate for 1–2 weeks. The differentiated and mature MPC5 cells were used in subsequent experiments. SV40-MES-13 cells were cultured in DMEM/F12 medium containing 10% FBS and 1% penicillin streptomycin. The cells were cultured in a 5% CO_2_ incubator at 37°C for standby.

### 2.6 Single gene GSEA pathway analysis

We performed Gene set enrichment analysis (GSEA) for mTOR and VDR using clusterProfiler, limma, ggplot2, enrichplot, and patchwork packages on dataset GSE142025, with *p* < 0.05 indicating statistical significance.

### 2.7 Cell transfection

Take MPC5 cells and SV40-MES-13 cells in logarithmic growth period, and adjust the cell density to 1 × 10^6^ cells/dish, inoculated in 10 cm culture dish, 10 mL for each dish. MPC5 and SV40-MES-13 cells in logarithmic growth were adjusted to a density of 1 × 10^6^ cells/dish and inoculated in 10 cm dishes with 10 mL medium each. When the cell fusion degree is 80%–90%, the basic culture medium (without added serum) is used to prepare the DDIT4 transfection complex according to the operating guide of Lipofectamine 2000 reagent. The transfection complex was added to MPC5 cells and SV40-MES-13 cells and replaced with complete medium after 6 h. MPC5 cells and SV40-MES-13 cells transfected with DDIT4 were harvested 24 h later.

### 2.8 Cell grouping

MPC5 and SV40-MES-13 cells were categorized into four groups: Control (5.5 mmol glucose and 19.5 mmol mannitol), HG (25 mmol glucose), HG + OE-NC (25 mmol glucose with blank plasmid), and HG + OE-DDIT4 (25 mmol glucose with DDIT4 plasmid).

### 2.9 Transmission electron microscope

Following 24 and 48 h post-treatment, MPC5 and SV40-MES-13 cells were prepared for observation under a transmission electron microscope. Centrifuge the cells at 3000 rpm at low speed to collect the precipitation. After PBS cleaning twice, add 2.5% glutaraldehyde to fix for 2–4 h. Cell samples were pre embedded in 3% low melting point agar, rinsed with 0.1 M phosphoric acid rinse solution for 3 times, and then placed in 1% osmic acid for 4°C fixation for 2 h. The samples were successively dehydrated in 30%, 50%, 70%, 80%, 95%, and 100% ethanol, and then 100% propylene oxide was added for 3 times. Penetrate the embedded sample with epoxy propane and the embedding solution at room temperature, and treat it at 60°C for 48 h to make the resin fully polymerized. The ultrathin microtome was used to slice at 70nm, and 3% uranium acetate saturated ethanol solution was used for staining for 8 min, then 2.7% lead citrate solution was used for staining for 8 min. Observe and take photos under the transmission electron microscope.

### 2.10 Laser confocal

MPC5 and SV40-MES-13 cells in logarithmic phase were adjusted to 0.8 × 10^4^ cells/dish and seeded in 96-well plates, with 100 μL per well. Cell transfection and grouping treatment are the same as 2.2.4 and 2.2.5. After 48 h of grouping treatment, MPC5 cells and SV40-MES-13 cells were collected for laser confocal microscope observation. Cells were fixed with 4% paraformaldehyde and stained with DAPI. Observe and take photos under laser confocal microscope.

### 2.11 qRT-PCR

MPC5 and SV40-MES-13 cells in the logarithmic growth phase were adjusted to a density of 2 × 10^5^ cells/dish and seeded into 12-well plates, with 1 mL medium per well. Cell transfection and grouping treatment are the same as 2.2.4 and 2.2.5. After 48 h of grouping treatment, MPC5 cells and SV40-MES-13 cells were collected for qRT-PCR detection. Each group of cells was added with corresponding amount of Trizol lysate to lyse cells and extract total RNA from cells. After the total RNA was reverse transcribed into cDNA, it was amplified by real-time fluorescence quantitative PCR. The total PCR reaction system is 20 µ L: SYBR Green Mix 10 µ L, upstream primer 0.4 µ L, downstream primer 0.4 µ L, dd H_2_O 7.2 µ L, cDNA template 2 µ L. PCR reaction conditions: 94°C pre denaturation for 10 min, 1 cycle; denatured at 94°C for 20s, annealed at 55°C for 20s, extended at 72°C for 20s, 40 cycles in total.

### 2.12 Western blotting

Cells from each group were lysed on ice using RIPA buffer containing 1% PMSF to extract total protein. The protein concentration was determined with BCA kit. The loading mass of each pore protein is 30 μ g. Sodium dodecyl sulfate (SDS) - polyacrylamide gel electrophoresis (PAGE) was used for electrophoresis, and the protein was transferred to PVDF membrane. After 5% BSA was sealed at room temperature for 1h, rabbit anti VDR (1:1000), rabbit anti mTOR (1:1000), rabbit anti p70s6k (1:1000), rabbit anti 4E-BP (1:1000), rabbit anti LC3II (1:1000), and rabbit anti LC3I (1:1000) antibodies were added respectively. After incubation at 4°C overnight, the secondary antibody corresponding to the primary antibody was added and incubated at room temperature for 2 h. ECL luminous solution develops color and collects images. GAPDH is the internal parameter.

### 2.13 ELISA

Cellular activities of malondialdehyde (MDA), superoxide dismutase (SOD), and glutathione (GSH) were measured using ELISA kits from Dojindo Company. All tests were carried out in strict accordance with the instructions of the kit.

### 2.14 Data analysis

Statistical analyses were performed using SPSS 22.0, with data presented as mean ± standard deviation (SD). The significance between multiple groups were determined by one-way variance (ANOVA) analysis and LSD *post hoc* multiple comparison test. *P* < 0.05 means the difference is statistically significant.

## 3 Results

### 3.1 Differential expression of mTOR and VDR in renal tissues of DKD patients: Upregulation of mTOR and downregulation of VDR

**FIGURE 1:**
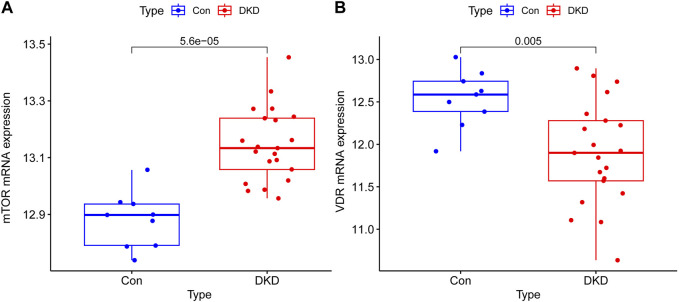
Transcriptional expression results of mTOR and VDR in renal tissue of Human. **(A)** The mRNA expressions of mTOR in renal tissue of DKD patients were overexpressed compared with control group and has a great significance (*p* < 0.001) **(B)** The mRNA expressions of VDR were decreased in DKD group and has a statistical significance compared with control group (*p* < 0.05).

The mRNA expressions of mTOR in renal tissue of DKD patients were overexpressed compared with control group and has a great significance (*p* < 0.001), the mRNA expressions of VDR were decreased in DKD group and has a statistical significance compared with control group (*p* < 0.05) (Figure 1).

### 3.2 Structural abnormalities and VDR-mTOR signaling pathway changes in DKD patient kidney samples

**FIGURE 2:**
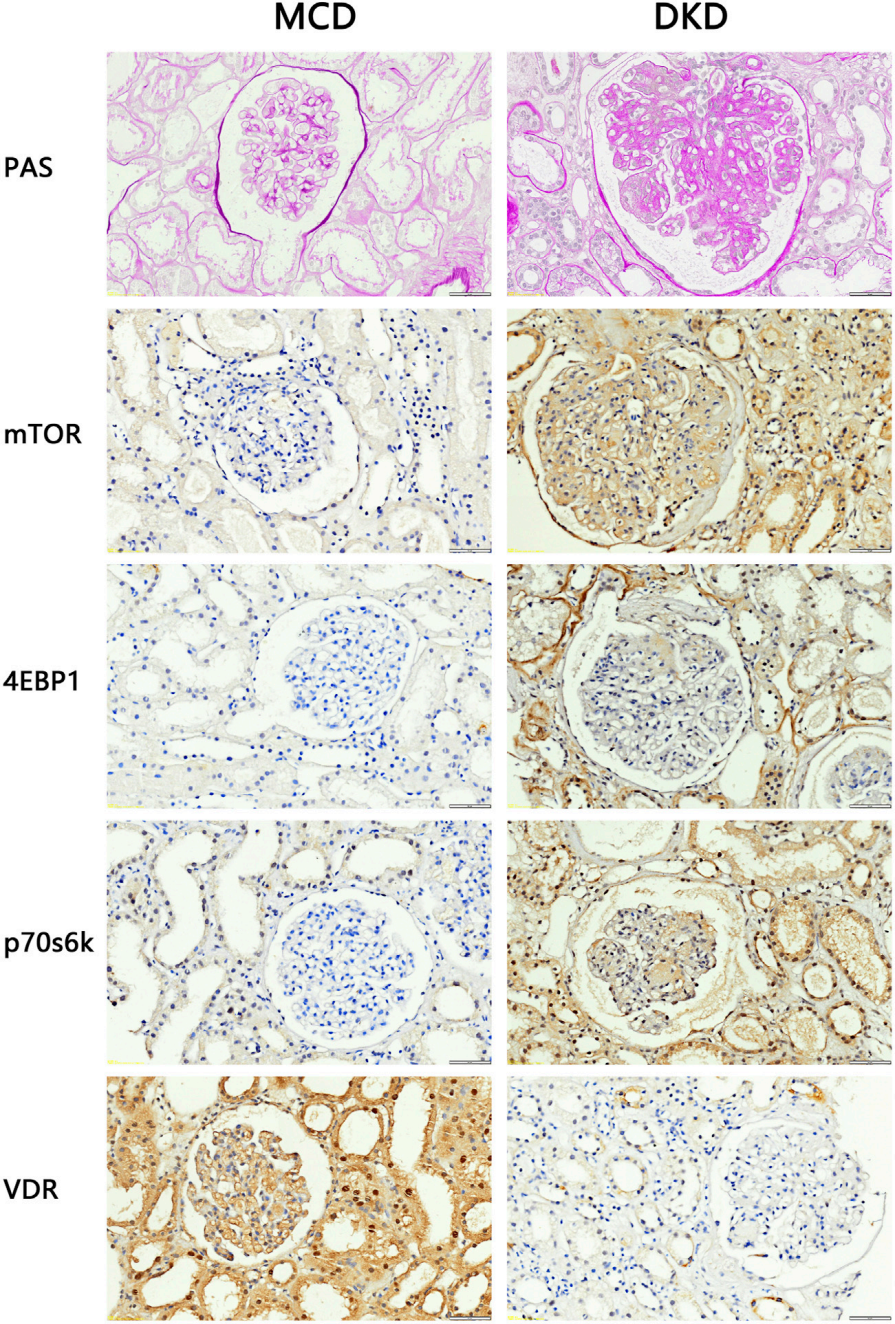
PAS staining and IHC staining results of clinical kidney samples (4QOx). The results of PAS staining showed that compared with patients with minimal change disease, patients with diabetic nephropathy had hyperplasia of glomerular mesangium, thickened base­ment membrane, hypertrophy of glomeruli, and formation of K-W nodules. The results of IHC staining showed that compared with MCD patients, the expression of VDR in the kidney tissues of DKD patients was reduced, and the expression of mTOR and downstream signaling pathway proteins was increased.

To investigate alterations in the VDR-mTOR pathway in human samples, clinical specimens were subjected to PAS staining and IHC staining. Consistent with the aforementioned findings, the average glomerular area and the relative area of glomerular extracellular matrix in DKD patients increased compared with MCD patients (Figure 2). According to the PAS scoring criteria, the glomerulosclerosis score of clinical DKD samples was significantly higher at 3.33 (n = 3) compared to the glomerulosclerosis score of MCD samples, which was 1.33 (n = 3) (*p* < 0.05). In addition, IHC results showed that the expression of mTOR, p70s6k, 4E-BP1 in DKD patients was significantly higher than that in MCD patients, and the expression of VDR was significantly lower than that in MCD patients (Figure 2). These results suggest that there is renal pathological damage in diabetes nephropathy, which may be related to mTOR/4E-BP1 signaling pathway.

### 3.3 Decline in renal function and metabolic abnormalities in DKD model mice

At the animal level biochemical markers were employed to investigate the progression of DKD in db/db mice. As shown in Table 1, the GFR of db/db model mice at 8 weeks of age (471.7 ± 75.8 μL/min) was higher than that of the m/m normal control mice (250.3 ± 60.7 μL/min). The GFR further increased significantly at 20 weeks of age. Urinary microalbumin in the db/db model group at 20 weeks of age (0.060 ± 0.014 mg) was significantly higher than in the m/m normal control group (0.010 ± 0.001 mg) (*p* < 0.05). Fasting blood glucose (FBG) in the db/db model group at 20 weeks of age (25.9 ± 6.5 mmol/L) was higher than in the m/m normal control group (7.0 ± 0.4 mmol/L) (*p* < 0.05). In conclusion, at the age of 20 weeks, db/db mice exhibited significant disparities in GFR, u-mAlb and FBG, demonstrating characteristic biochemical alterations associated with DKD.

**TABLE 1:** GFR, urinary microalbumin, and fasting blood glucose results of mice at different ages.

	8w (n = 6)	20w (n = 6)
GFR (uL/min)		
db/db	471.7 ± 75.8	774.1 ± 90.3
m/m	250.3 ± 60.7	209.1 ± 37.4
u-mAlb(mg)		
db/db	0.020 ± 0.008	0.060 ± 00.014*
m/m	0.010 ± 0.008	0.010 ± 0.001
FBG (mmol/L)		
db/db	11.5 ± 3.7	25.9 ± 6.5*
m/m	5.1 ± 1.3	7.0 ± 0.4

Given concerns about the potential influence of body size on GFR measurements, it is crucial to consider that while db/db mice generally have a larger body size, the pronounced increase in GFR and altered urine volume primarily reflect the underlying diabetic pathology rather than size differences alone. This study’s findings, including the differential urine volume collected over 12 h for albumin measurements, underscore the diabetic nephropathy model’s validity in db/db mice, revealing significant biochemical markers indicative of DKD.

### 3.4 Kidney damage and VDR-mTOR signaling pathway variations in DKD model mice

**FIGURE 3:**
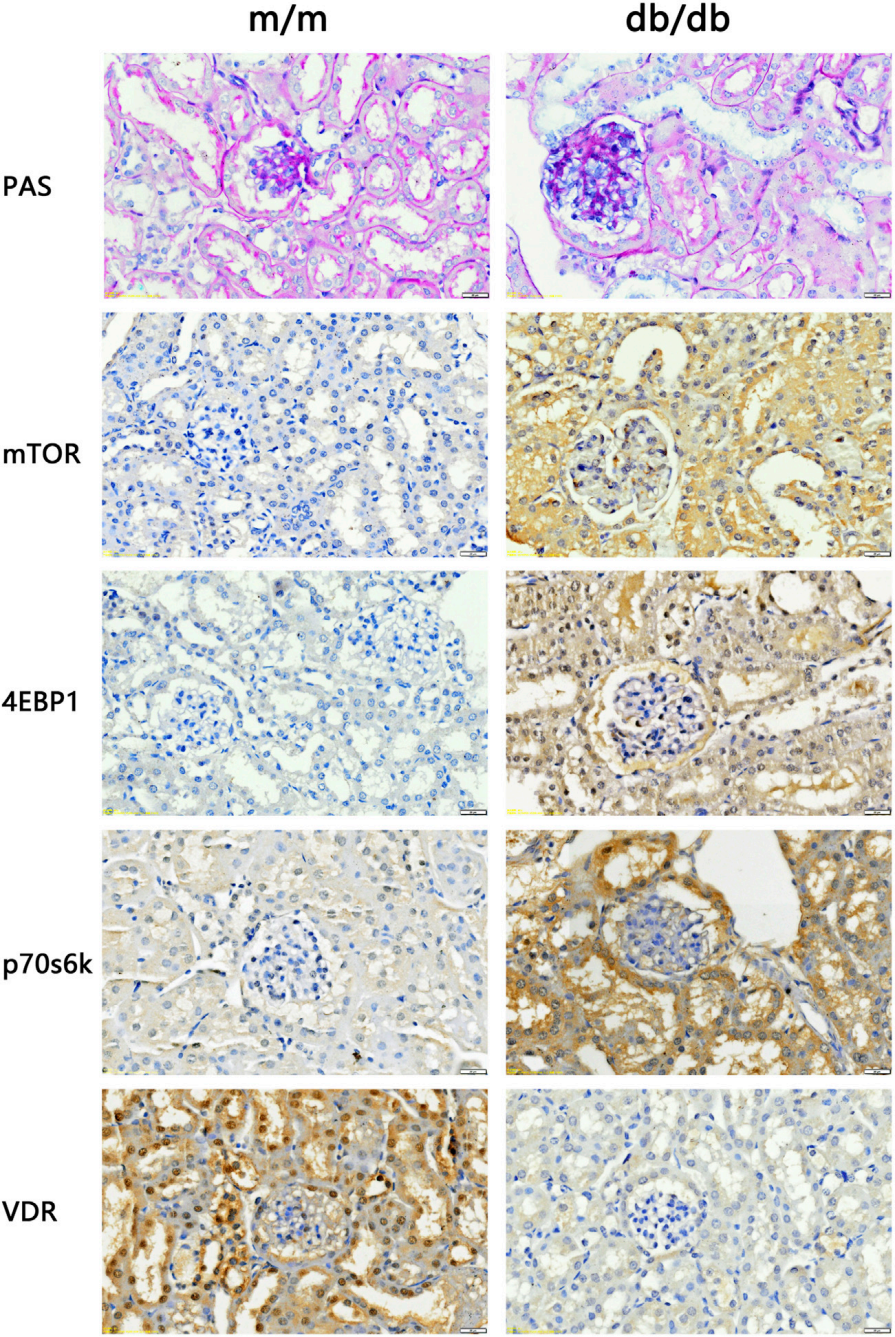
PAS staining and IHC staining results of kidney tissue of experimental mice (400x). The results of PAS staining showed no abnormal pathological changes in the kidney structure of the 20 weeks old m/m mice. Compared with m/m mice, db/db mice at the same age had hyperplasia of glome­ rular mesangium, thickened basement membrane, and hypertrophy of glomeruli. IHC staining results showed that the expression of VDR in the kidney tissue of db/db mice was reduced, and the expression of mTOR and downstream signaling pathway proteins was increased.

The renal tissues of the experimental mice were stained with PAS. The results showed that the capillary loops of the glomerulus of the 20 weeks old m/m mice were thin and clear, and there were no abnormal pathological changes in the renal structure. Compared with m/m mice, there were irregular nodular PAS positive material deposits in the glomeruli of 20 weeks old db/db mice, glomerular mesangial matrix hyperplasia, and basement membrane thickening (Figure 3). The results confirmed the successful construction of the DKD animal model, with characteristic pathological changes observed in kidney tissue of 20-week-old db/db mice. In the DKD model group, db/db mice exhibited a PAS glomerulosclerosis score of 3.66 (n = 3), which was significantly higher than the glomerulosclerosis score of 1.33 (n = 3) observed in the control group m/m samples (*p* < 0.05). In addition, IHC results showed that compared with m/m mice, the expression of mTOR, p70s6k, 4E-BP1 in db/db mice was significantly increased, and the expression of VDR was significantly reduced (Figure 3). The findings are consistent with the results of bioinformatics analysis, indicating a significant perturbation in the VDR-mTOR pathway in DKD.

### 3.5 High glucose induces significant organelle damage in podocytes and mesangial cells

**FIGURE 4:**
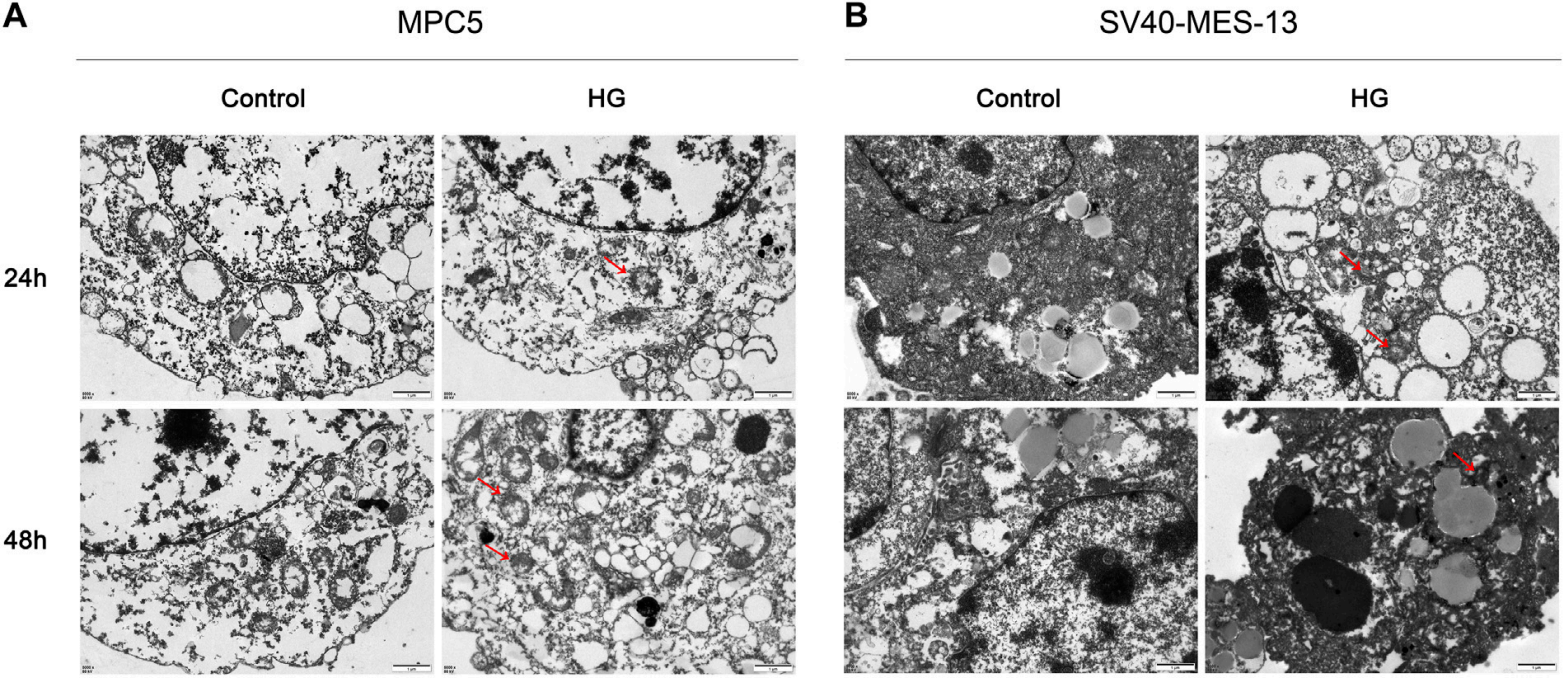
Transmission electron microscope observation results of MPC5 cells and SV40-MES-13 cells (500Qx). **(A)** The results of transmission electron microscopy in MPC5 cells. **(B)** The results of transmission electron microscopy in SV40-MES-13 cells.

In order to simulate DKD injury and further elucidate the regulatory mechanism of the VDR-mTOR pathway, as well as develop appropriate regulatory strategies, we established an *in vitro* cell model of DKD using high glucose culture conditions. The cellular and substructural differences were subsequently examined through transmission electron microscopy (TEM). The transmission electron microscopy results revealed that MPC5 and SV40-MES-13 cells exposed to high glucose exhibited significant mitochondrial swelling, ridgeline dissolution, vesicular expansion of endoplasmic reticulum compared to the control group. The findings suggest that podocyte and mesangial cells undergo damage in a high glucose environment. Moreover, these changes were more pronounced at 48 h than at 24 h (Figure 4). The results showed that podocytes and mesangial cells were damaged by high glucose.

### 3.6 Visualization of DDIT4 plasmid transfection efficiency

**FIGURE 5:**
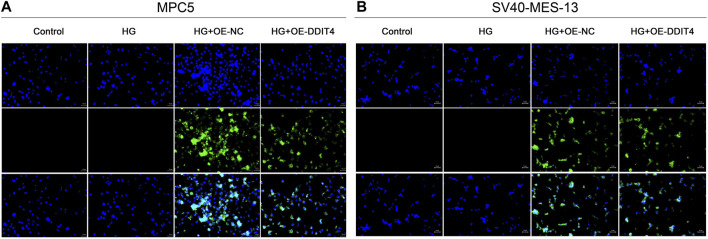
Laser confocal results of MPC5 cells and SV40-MES-13 cells. **(A)** Laser confocal results in MPC5 cells. **(B)** Laser confocal results in SV40-MES-13 cells.

To investigate the protective effect of DDIT4 on a cell model of diabetic kidney disease (DKD) and its regulatory role in the VDR-mTOR pathway, we performed cell transfection experiments using an EGFP-tagged DDIT4 plasmid and utilized laser confocal microscopy to visualize the transfection efficiency. Laser confocal results showed that in MPC5 cells and SV40-MES-13 cells, compared with Control group and HG group, HG + OE-NC group and HG + OE-DDIT4 group had obvious green fluorescence, indicating that plasmid transfection cells were successful (Figure 5). In addition, the expression of green fluorescence in the HG + OE-DDIT4 group was lower than that in the HG + OE-NC group (Figure 5), which confirmed that DDIT4 may participate in the pathogenesis of diabetic kidney disease and improve the pathological damage of diabetic kidney disease. The green fluorescence observed in the HG + OE-DDIT4 and HG + OE-NC groups serves as a marker of transfection efficiency, demonstrating successful delivery of the DDIT4 plasmid into cells. The reduced fluorescence intensity in the HG + OE-DDIT4 group compared to the HG + OE-NC group suggests not only successful transfection but also a subsequent cellular response, potentially indicative of DDIT4 exerting its protective effects in a high-glucose environment.

### 3.7 DDIT4 promotes recovery and growth of damaged cells

**FIGURE 6:**
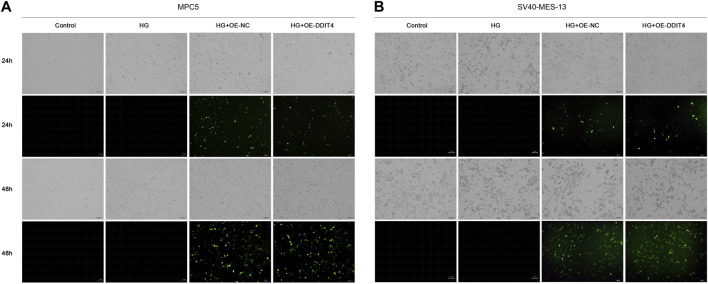
MPC5 cells and SV40-MES-13 cells modeling and transfection. **(A)** The results of cell transfection in MPC5 cells. **(B)** The results of cell transfection in SV40-MES-13 cells.

Light field cell filming showed that MPC cells and SV40-MES-13 cells cultured in high glucose had significant damage compared with normal cells, and DDIT4 transfected cells cultured in high glucose had mild damage. As can be seen from the fluorescence map, the plasmid was successfully transferred into the cells, and the transfection effect was better at 48 h (Figure 6).

### 3.8 DDIT4 modulates the VDR-mTOR pathway to counteract high glucose-induced damage

**FIGURE 7:**
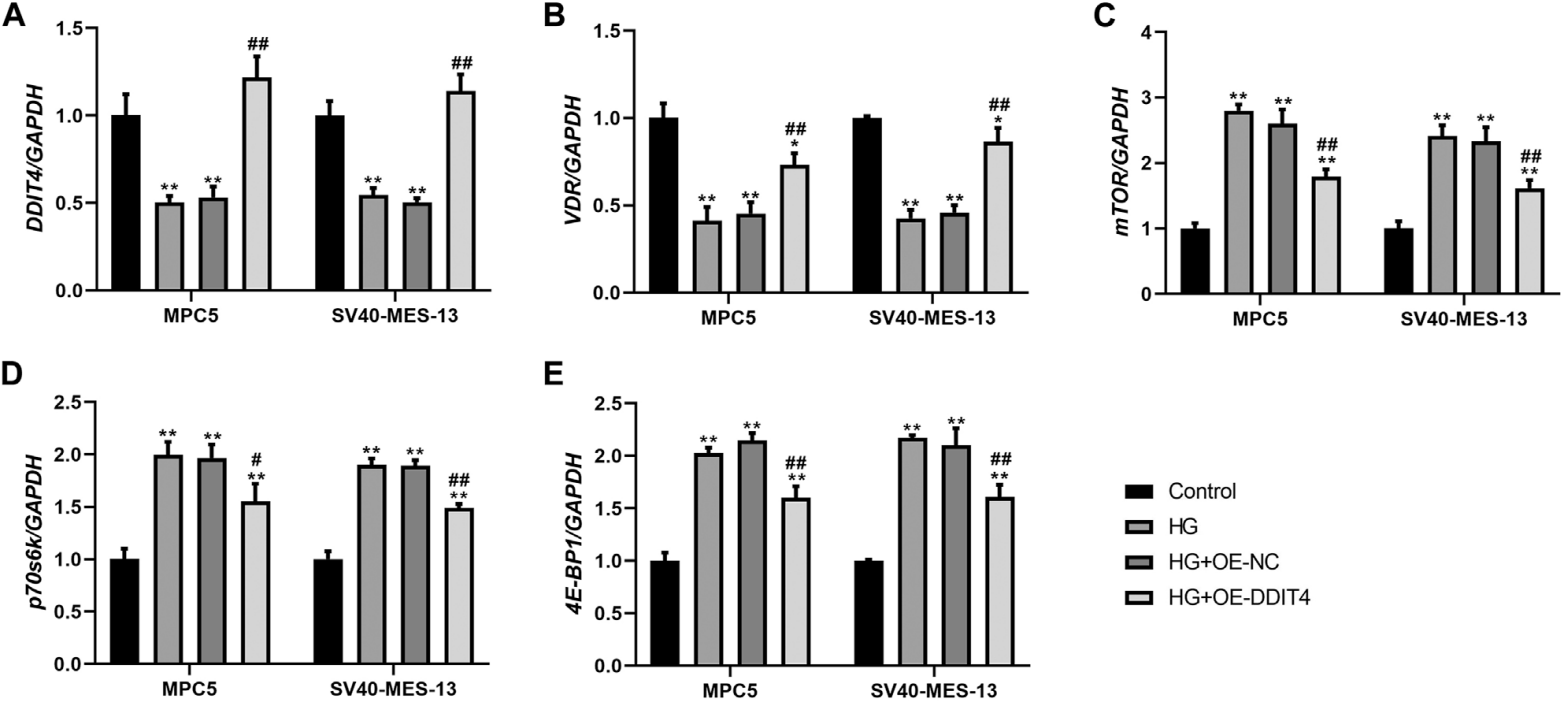
qRT-PCR results of MPC cells and SV40-MES-13 cells. **(A)** The expressions of DDIT4. **(B)** The expressions of VDR. **(C)** The expressions of mTOR. **(D)** The expressions of p70s6k. **(E)** The expressions of 4E-BP1. ∗∗ p < 0.01, compared with Control group; #*p* < 0.05, *##p*<0.01, compared with HG group. The experiments were independently repl cated three times to ensure the reproducibility and accuracy.

**FIGURE 8:**
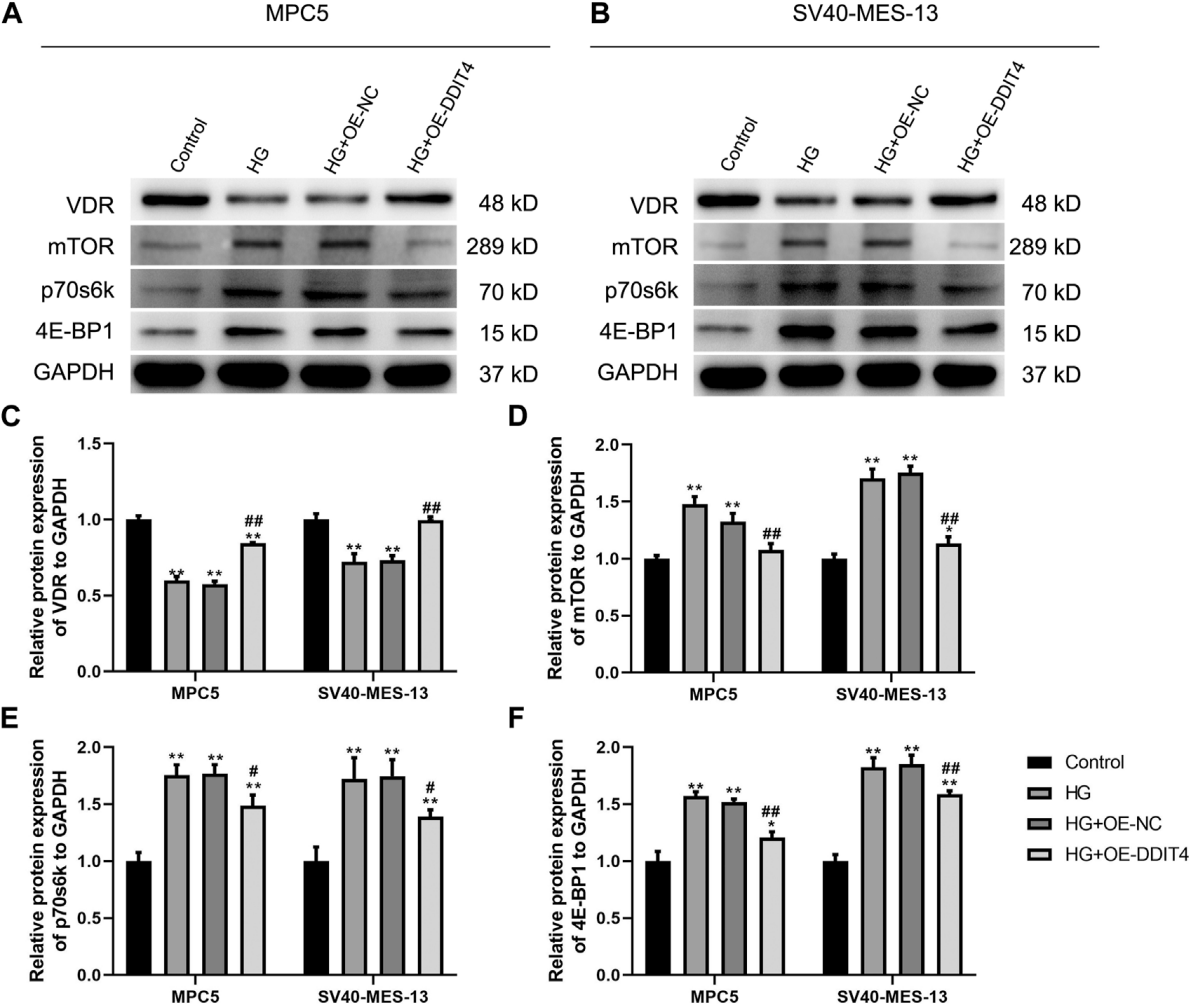
**(A)** Western Blotting for VDR/mTOR/p70s6k/4E -BP1 protein expression in MPC cells and SV40-MES-13 cells. **(A)** Western blots of protein expressionin MPC5 cells as indicated. **(B)** Western blots of protein expression in SV40-MES·13 cells as indicated. **(C)** The relative protein expression of VDR to GAPOH. **(D)** The relat ve protein expression of mTOR to GAPOH. **(E)** The relative protein expression of p70s6k to GAPDH. **(F)** The relative protein expression of 4E-BP1 to GAPOH. ∗p < 0.05, ∗∗p < 0.01, compared with Control group; #p < 0.05,##p<0.01, compared with HG group. The experiments were independently replicated three times to ensure the reproducibility and accuracy.

To investigate the expression of the VDR-mTOR pathway in a cellular model of DKD and elucidate the regulatory mechanism of DDIT4 on this pathway, we employed quantitative real-time PCR (qRT-PCR) and Western Blotting (WB) to assess both the gene and protein expression within the pathway. The results of qRT-PCR showed that in MPC5 cells and SV40-MES-13 cells, the expressions of DDIT4 and VDR in HG group were significantly lower than those in Control group (*p* < 0.01), while the expressions of mTOR, p70s6k and 4E-BP1 were significantly higher than those in Control group (*p* < 0.01) (Figure 7); Compared with HG group, the expression of DDIT4 and VDR in HG + OE-DDIT4 group increased significantly (*p* < 0.01), while the expression of mTOR, p70s6k, 4E-BP1 decreased significantly (*p* < 0.01, *p* < 0.05) (Figure 8). DDIT4 is an important target of VDR/mTOR/p70s6k/4E-BP1 signaling pathway induced by high glucose in diabetic nephropathy.

### 3.9 DDIT4 enhances autophagy by modulating LC3I and LC3II expression

**FIGURE 9:**
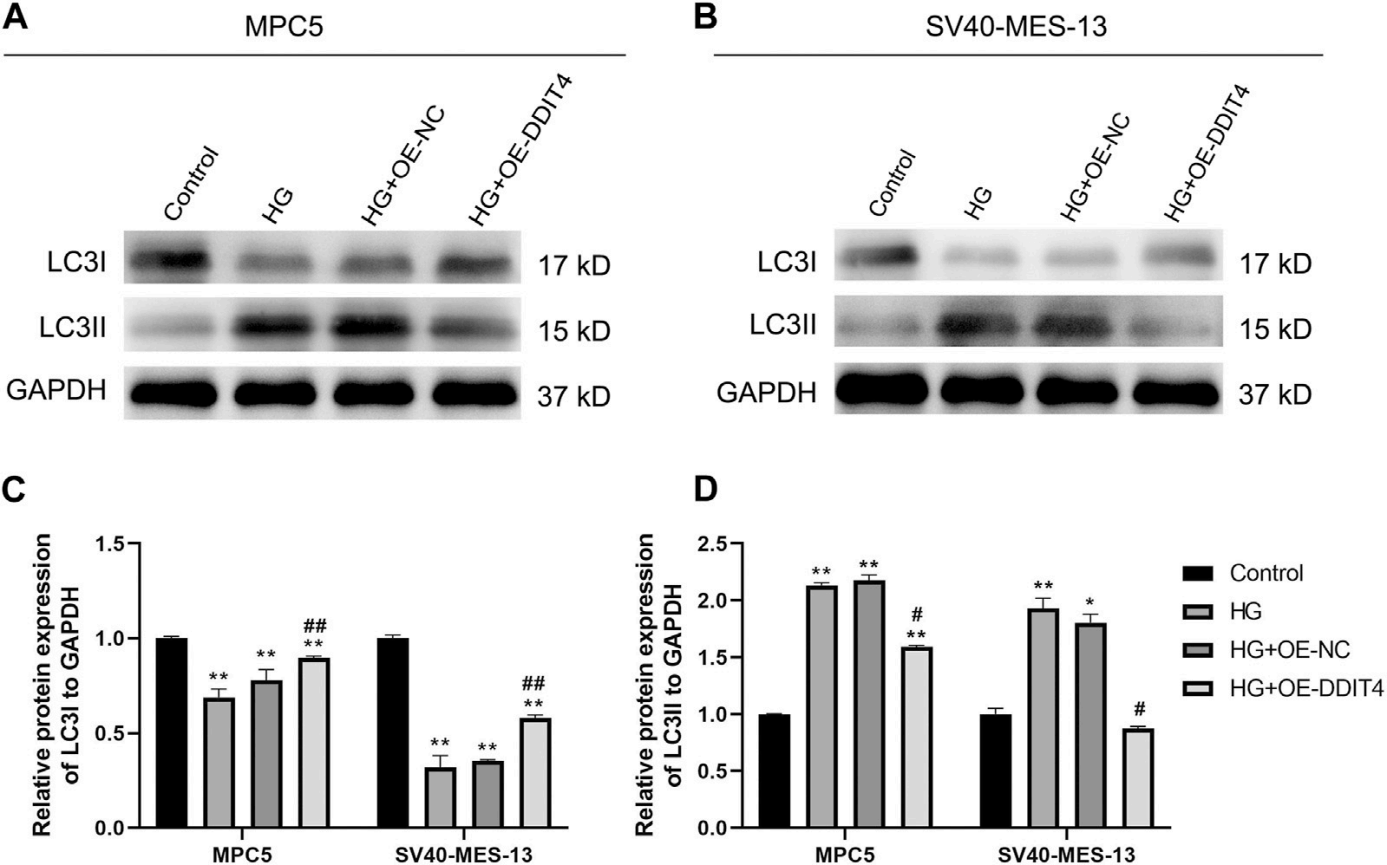
Western Blotting for LC31 and LC311 protein expression in MPC cells and SV40-MES-13 cells. **(A)** Western blots of protein expression in MPC5 cells asindicated. **(B)** Western blots of protein expression in SV40-MES-13 cells as indicated. **(C)** The relative protein expression of LC31to GAPDH. **(D)** The relative protein expression of LC311 to GAPDH. ∗ *p* < 0.05, ∗∗ *p* < 0.01, compared with Control group; #*p* < 0.05, ##p < 0.01, compared with HG group. The experiments were independently replicated three times to ensure the reproducibility and accuracy.

To further elucidate the underlying mechanism of DDIT4’s impact on cellular damage, we conducted an investigation into the expression levels of autophagy marker proteins LC3I and LC3II. Our results showed that compared to the Control group, HG group MPC cells and SV40-MES-13 cells had significantly decreased expression of LC3I (*p* < 0.01) but increased expression of LC3II (*p* < 0.01). However, after OE-DDIT4 treatment under high glucose conditions, there was a significant increase in LC3I expression relative to HG group (*p* < 0.01), accompanied by a significant decrease in LC3II levels (*p* < 0.05), indicating that DDIT4 enhances autophagy in a high glucose environment (Figure 9).

### 3.10 GSEA pathway anslysis reveals DDIT4-regulated mechanisms in VDR and mTOR signaling

**FIGURE 10:**
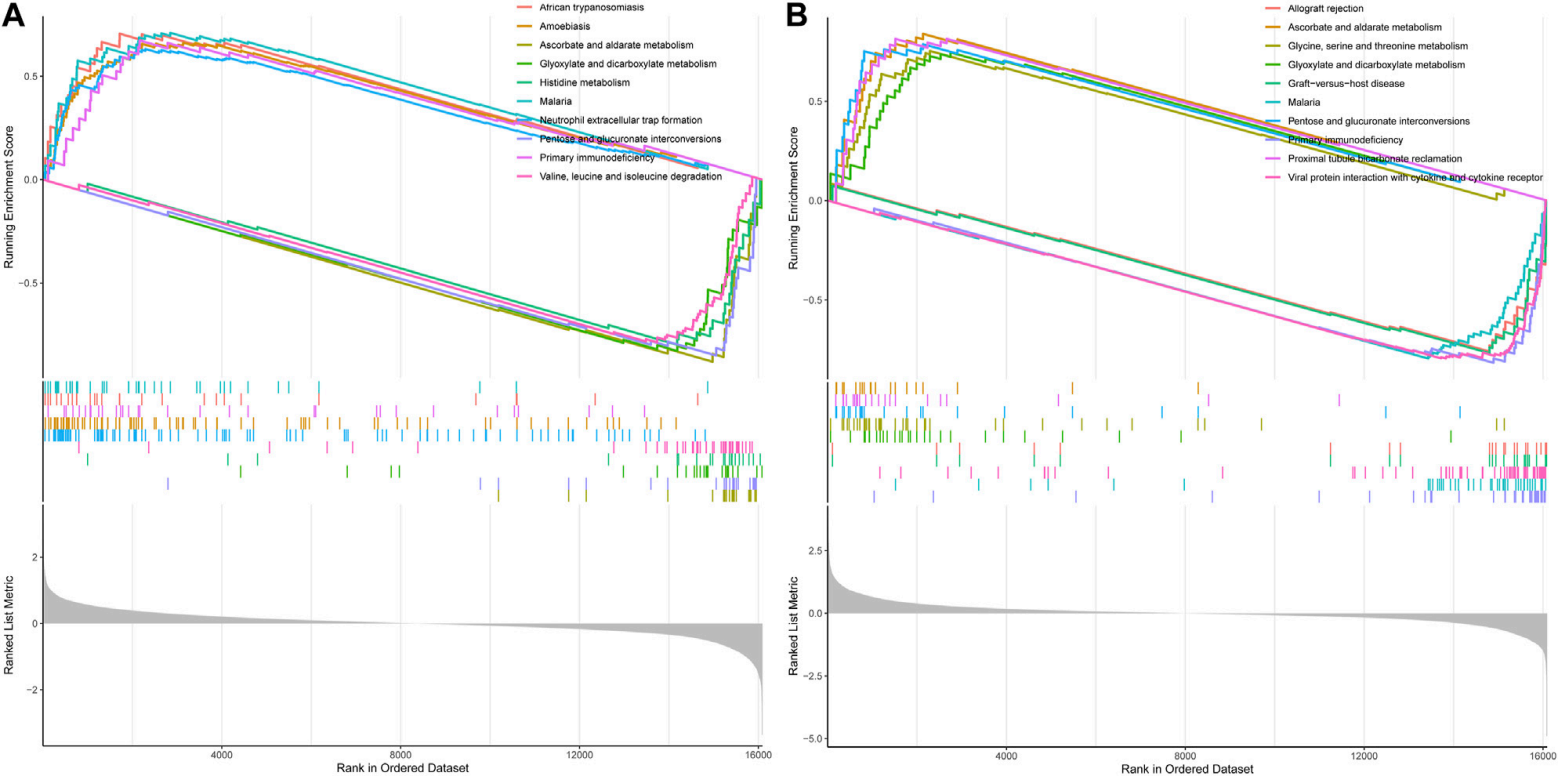
GSEA pathway analysis of mTOR and VDR. **(A)** GSEA of mTOR **(B)** GSEA of VOR. Both mTOR and VDR GSEA pathway analysis wereinvolvedinoxidative stress.

Next, we performed GSEA pathway analysis to enrich the associated pathways of VDR and mTOR based on the differential expression of genes between high and low expression groups. In the GSEA analysis of mTOR, several significant pathways emerged, including ascorbate and aldarate metabolism, histidine metabolism, and neutrophil extracellular trap formation, among others, which differentiated the high mTOR expression group from the low mTOR expression group (Figure 10A).

Similarly, in the GSEA analysis of VDR, notable enriched pathways encompassed ascorbate and aldarate metabolism, glycine, serine, and threonine metabolism, glyoxylate and dicarboxylate metabolism, pentose, and glucuronate interconversions, as well as proximal tubule bicarbonate reclamation, distinguishing the high VDR expression group from the low VDR expression group (Figure 10B). Both mTOR and VDR GSEA pathway analyses highlighted essential biochemical processes influenced by DDIT4.

### 3.11 DDIT4 modulates oxidative stress levels through VDR/mTOR/p70s6k/4E-BP1 signaling pathway

**FIGURE 11:**
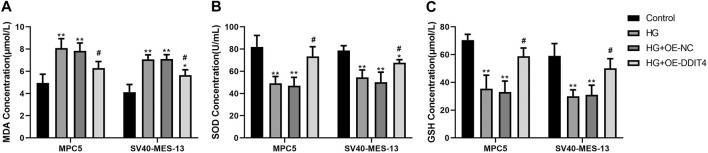
ELISA results of MPC cells and SV40-MES-13 cells. **(A)** MDA Concentration.**(B)** SOD Concentration. **(C)** GSH Concentration. The experiments were independently replicated three times to ensure the reproducibi ty and accuracy. ∗∗ p < 0.01, compared with Controlgroup; #p < 0.05, compared with HG group.

To establish the association with cellular oxidative stress levels, the oxidative stress-related markers MDA, SOD, and GSH were quantified using ELISA methodology. The ELISA results demonstrated a significant increase in MDA expression (*p* < 0.01) and a significant decrease in SOD and GSH expression (*p* < 0.01) in both MPC cells and SV40-MES-13 cells when compared to the control group. Furthermore, the HG + OE-DDIT4 group exhibited a significant reduction in MDA expression (*p* < 0.05) along with a notable increase in SOD and GSH expression (*p* < 0.05), as compared to the HG group (Figure 11). The findings are consistent with the results of gene set enrichment analysis (GSEA), indicating that DDIT4 may modulate cellular oxidative stress levels through involvement in the VDR/mTOR/p70s6k/4E-BP1 signaling pathway, thereby ameliorating pathological damage associated with diabetic nephropathy.

## 4 Discussion

The development of DKD is multifaceted, encompassing glucose metabolism disturbances, hemodynamic alterations, cytokine expression, oxidative stress, inflammation, and immune responses. (Thomas et al., 2015; Tang and Yiu, 2020). Due to the complex pathogenesis of DKD involving numerous factors, there is currently no specific method for prevention and treatment (Sharov et al., 2022). The characteristic pathological features of DKD include diffuse thickening of glomerular capillary basement membrane, proliferation of mesangial cells and mesangial matrix, and then nodular sclerosis (Thomas, 2021). The destruction of glomerular filtration membrane structure is the common mechanism underlying various glomerular diseases characterized by proteinuria. Podocytes, key components of the glomerular filtration membrane, are significantly correlated with DKD’s onset and progression (Lassén et al., 2022; Zang et al., 2022). Under normal conditions, podocytes show increased autophagic activity, facilitating organelle renewal and self-metabolism. However, in diabetic patients, detrimental factors such as elevated glucose levels and metabolites collaborate with podocytes often resulting in the accumulation of damaged proteins and organelles in cells (Fu et al., 2020). Failure to clear these substances promptly leads to cytotoxicity and an irreversible decrease in viable cell count. (Dai et al., 2017). The glomerulus serves as the characteristic site of renal pathological damage in DKD disease. The 2010 pathological staging system for DKD established by the Society of Renal Pathology primarily focuses on glomerular disorders (Tervaert et al., 2010). In this study, db/db mice were utilized to establish an animal model of diabetic kidney disease (DKD). The results of glomerular filtration rate (GFR), urinary microalbuminuria, and fasting blood glucose levels were consistent with the expected changes in biochemical indicators of DKD and significantly differed from those observed in control m/m mice. Kidney tissue samples from 20-week-old db/db mice, m/m mice, as well as patients with minimal change disease (MCD) and DKD underwent PAS staining to observe significant physiological changes. Immunohistochemical staining was performed to analyze the expressions of VDR, mTOR, p70s6k, and 4E-BP1 proteins in the VDR-mTOR pathway. Our findings suggest that renal pathological injury associated with DKD may be linked to the mTOR/4E-BP1 signaling pathway, the result is consistent with previous studies.

Recent research has shown VDR inhibits the mTOR signaling pathway by enhancing DDIT4 expression, thereby hindering DKD progression (Yang et al., 2015; Shi et al., 2022). mTOR, a serine/threonine kinase, is crucial for regulating cell growth, metabolism, and autophagy. There are two different types of mTOR complexes, mTOR complex 1 (mTORC 1) and mTOR complex 2 (mTORC 2) (Syed et al., 2012). mTORC 2 is closely related to actin cytoskeleton, cell polarity and phosphorylated protein kinase B (AKT). mTORC 1 is activated by the phosphatidylinositol 3 kinase (PI3K) system. PI3K can activate AKT (a serine/threonine kinase). After AKT activation, it inhibits the expression of tuberous sclerosis protein 1/2 complex (TSC1/2), thereby promoting the expression of a rich homologue of Ras (Rheb) in the brain, ultimately leading to the activation of mTORC 1, and then phosphorylating two downstream proteins p70s6k and 4E-BP1, leading to increased ribosome and protein synthesis (Aoki and Fujishita, 2017; Chen et al., 2018b; Chen et al., 2022). Through the above signal pathways, mTOR regulates the progress of cell cycle and plays a role in regulating cell growth, metabolism and autophagy. High glucose and certain cytokines activate the mTOR signaling via the PI3K pathway, promoting cell proliferation and protein synthesis. (Yang et al., 2018). The mTOR complex is the receptor of amino acid, glucose and energy nutritional status, which can sense and transmit external stimulus signals in a timely manner. As the junction of multiple signal pathways in cells and the central signal factor, mTOR regulates cell growth cycle and nutrient metabolism by integrating signals. It was found that the expression of mTOR in renal tissues of patients with glomerular disease and animal models was significantly increased mTOR plays a gating role in autophagy (Saxton and Sabatini, 2017; Herb and Schramm, 2021; Tang et al., 2022). After activation, mTOR can phosphorylate p70s6k and 4E-BP1 in its downstream, and participate in regulating cell apoptosis and energy metabolism. In addition, the study also confirmed that in rat mesangial cell lines cultured in high glucose environment, mTOR regulates cell growth and proliferation by phosphorylating downstream proteins p70s6k and 4E-BP1, and DDIT4 inhibits cell proliferation by up regulating the expression of mTOR by up regulating TSC1/TSC2 complex (Chen et al., 2018a; Herb and Schramm, 2021). In osteoblasts, 1.25(OH)_2_D_3_ can inhibit cell proliferation, and DDIT4 is the direct target of 1.25(OH)_2_D_3_ (Wang et al., 2016). In β-cells, high glucose can downregulate the expression of DDIT4 in cells, and 1.25(OH)_2_D_3_ can upregulate the expression of DDIT4 in high glucose environment (Yang et al., 2015). 1.25(OH)_2_D_3_ can effectively inhibit the proliferation of mesangial cells through the DDIT4/TSC2/mTOR signal pathway (Wang et al., 2016). In our study, MPC5 renal podocyte and SV40-MES-13 mesangial cell lines were cultured in high glucose to create a diabetic cell model. Cell damage induced by high glucose was observed using both light microscopy and transmission electron microscopy. Subsequently, DDIT4 plasmid transfection was employed as a treatment and regulatory method to investigate the effects of transfection on cellular damage and downstream pathways. The results of qRT-PCR and western blotting demonstrated alterations in proteins associated with the VDR/mTOR/p70s6k/4E-BP1 pathway, confirming that DDIT4 enhances the pathological damage of DKD by participating in VDR/mTOR/p70s6k/4E-BP1 signal pathway, similar to the effect of 1.25(OH)_2_D_3_ on mesangial cells.

**FIGURE 12:**
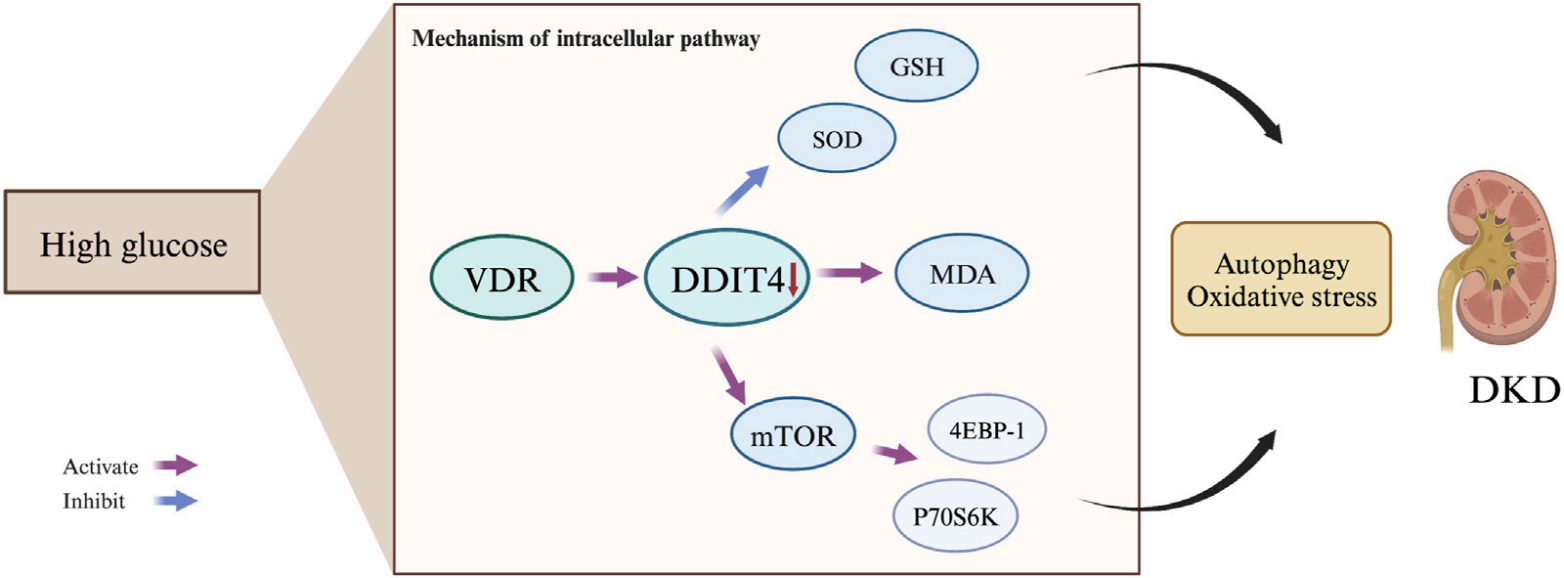
Mechanism of the VDR-mTOR Signaling Pathway in DKD Development. The VDR-mTOR signaling pathway plays a pivotal role in the progression of diabetic kidney disease {DKD). Under high glucose conditions, VDR expression is suppressed, resulting in reduced DDIT4 expression. Consequently, this promotes the expression of mTOR and its downstream signaling molecules, p70s6k and 4EBP-1. Simultaneously, it diminishes levels of the antioxidant enzymes SOD and GSH, while increasing the production of the lipid peroxidation product MDA. These molecular changes ultimately mediate autophagy. contributing to the initiation and progression of DKD.

Autophagy, a lysosome-mediated degradation process, selectively eliminates damaged proteins and organelles to maintain cellular homeostasis and supports self-recycling under stress. Oxidative stress occurs when there is an imbalance between oxidation and antioxidant systems, resulting in cellular damage. Accumulating evidence indicates that reactive oxygen species (ROS) generated during oxidative stress play a significant role in inducing autophagy. Conversely, autophagy acts as a protective mechanism by mitigating oxidative damage and promoting cell survival. Recently, the discovery of ferritinophagy as a selective type of autophagy has inspired further exploration into functional interactions between metabolism, immunity, and cell death (Liu et al., 2020). Recent studies exhibit that mTORC1 neutralizes ferroptosis through promoting GPX4 protein synthesis and/or inhibiting autophagy (Zhang et al., 2021). Our study demonstrates the critical role of the VDR/mTOR/p70s6k/4E-BP1 signaling pathway in DKD pathogenesis. LC3 (Microtubule-associated proteins 1A/1B light chain 3) serves as an autophagic marker protein, with LC3I and LC3II being involved in autophagosome membrane elongation, fusion, and delivery processes while maintaining oxidative stress homeostasis *in vivo*. During the course of autophagy, the soluble form of LC3 (LC3-I), which does not associate with membranes, is transformed into the membrane-associated form LC3-II through its conjugation with phosphatidylethanolamine (PE). LC3-II then binds to the mature autophagic membranes, facilitating an increase in the formation of autophagosomes and consequently enhancing autophagy. This transformation is catalyzed by an amide bond formation with the C-terminal of LC3 through the action of the E2-like enzyme Atg3, and is mediated by an acyl transfer reaction catalyzed by Atg7 and Atg3. As a result of the conversion of LC3-I to LC3-II, a dynamic equilibrium is established that leads to a decrease in LC3-I protein levels and an increase in LC3-II protein levels. The GSEA pathway analysis revealed the involvement of both mTOR and VDR in the response to oxidative stress. Intriguingly, our results suggest a dynamic interplay between VDR and DDIT4, traditionally viewed as upstream of VDR. The observed increase in VDR expression upon DDIT4 transfection points to a potential feedback mechanism. This may imply that DDIT4 upregulation could indirectly enhance VDR expression, indicative of a compensatory cellular response to the high-glucose environment. This complex regulatory network, hinted at by the bidirectional influences of DDIT4 and VDR, underscores the need for further investigation to fully elucidate these interactions and their implications for DKD pathogenesis. MDA, as a marker of lipid peroxidation, along with SOD and GSH, as key antioxidative enzymes, provide insights into the cellular redox state. The reduction of oxidative stress through DDIT4-mediated pathways aligns with our observation of enhanced autophagy, further reinforcing the potential of DDIT4 as a therapeutic target. By detecting MDA, and noting the activities of SOD and GSH, we have delineated the antioxidative benefits of DDIT4 overexpression, which supports its role in mitigating the pathological damage associated with diabetic nephropathy (Figure 12).

## 5 Conclusion

In conclusion, exploring DDIT4’s role in the VDR-mTOR pathway uncovers a promising therapeutic avenue for DKD treatment. The observed changes in the VDR-mTOR pathway in both clinical samples and experimental models highlight its significance in the pathogenesis of DKD. The ability of DDIT4 to modulate key components of this pathway, enhance autophagy, and alleviate oxidative stress reveals its potential as a novel drug discovery target for DKD. These findings open up new avenues for innovative approaches to chronic kidney disease drug discovery. Further exploration and validation of the therapeutic potential of DDIT4 may offer effective interventions, meeting unmet clinical needs in DKD management.

## Data Availability

Publicly available datasets were analyzed in this study. This data can be found here: Transcriptional expression levels of mTOR and VDR in human renal tissue were obtained from the Gene Expression Omnibus Database (GSE142025).
