# Supplementary material for: Unraveling DDIT4 in the VDR-mTOR pathway: a novel target for drug discovery in diabetic kidney disease
Source: Front Pharmacol. 2024 Mar 19;15:1344113. doi: 10.3389/fphar.2024.1344113 (PMC10985261; doi:10.3389/fphar.2024.1344113)
Supplement: Supplementary file 1 [file DataSheet3.ZIP › Figure1 and 10data and R code/Figure10 data and Rcode/2.VDR_up_GSEA.pdf]

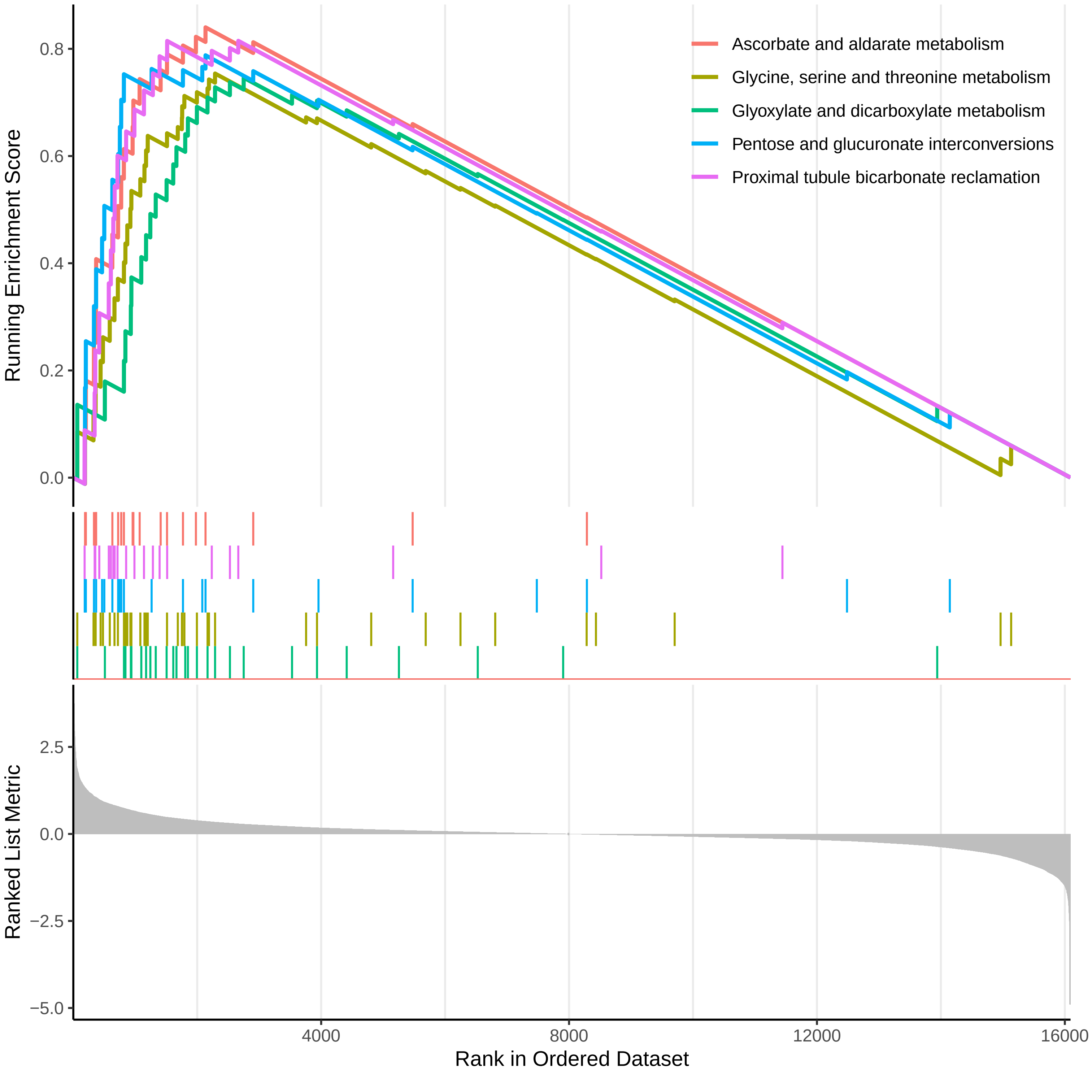

Running Enrichment Score
0.8
0.6
0.4
0.2
0.0
Ascorbate and aldarate metabolism
Glycine, serine and threonine metabolism
Glyoxylate and dicarboxylate metabolism
Pentose and glucuronate interconversions
Proximal tubule bicarbonate reclamation
Ranked List Metric
2.5
0.0
−2.5
−5.0
4000
8000
12000
16000
Rank in Ordered Dataset
