# Supplementary figures and images for: Unraveling DDIT4 in the VDR-mTOR pathway: a novel target for drug discovery in diabetic kidney disease
Source: Front Pharmacol. 2024 Mar 19;15:1344113. doi: 10.3389/fphar.2024.1344113 (PMC10985261; doi:10.3389/fphar.2024.1344113)

Type    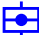 Con    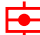 DKD

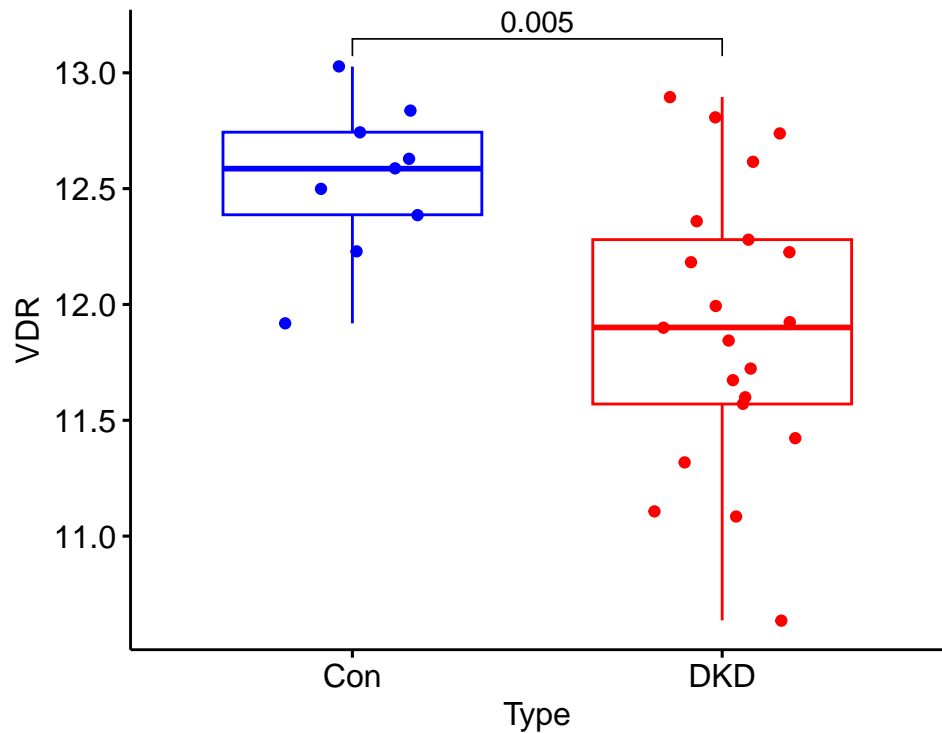

Supplement: Supplementary file 1 [file DataSheet3.ZIP › Figure1 and 10data and R code/Figure1 data and R code/VDR.pdf]

Type 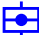 Con 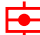 DKD

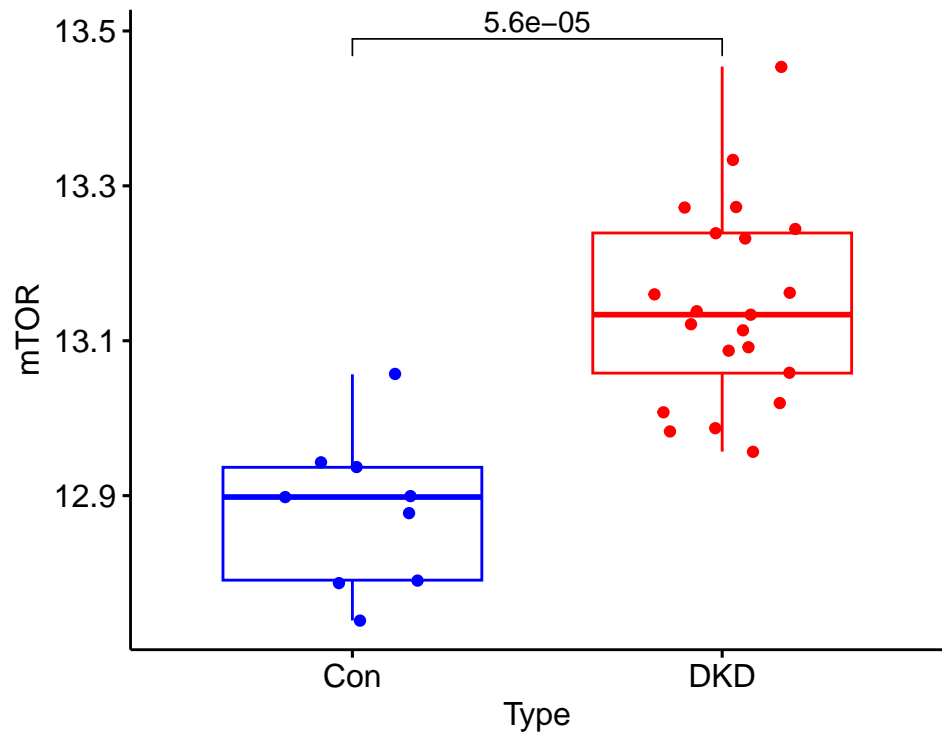

Supplement: Supplementary file 1 [file DataSheet3.ZIP › Figure1 and 10data and R code/Figure1 data and R code/mTOR.pdf]

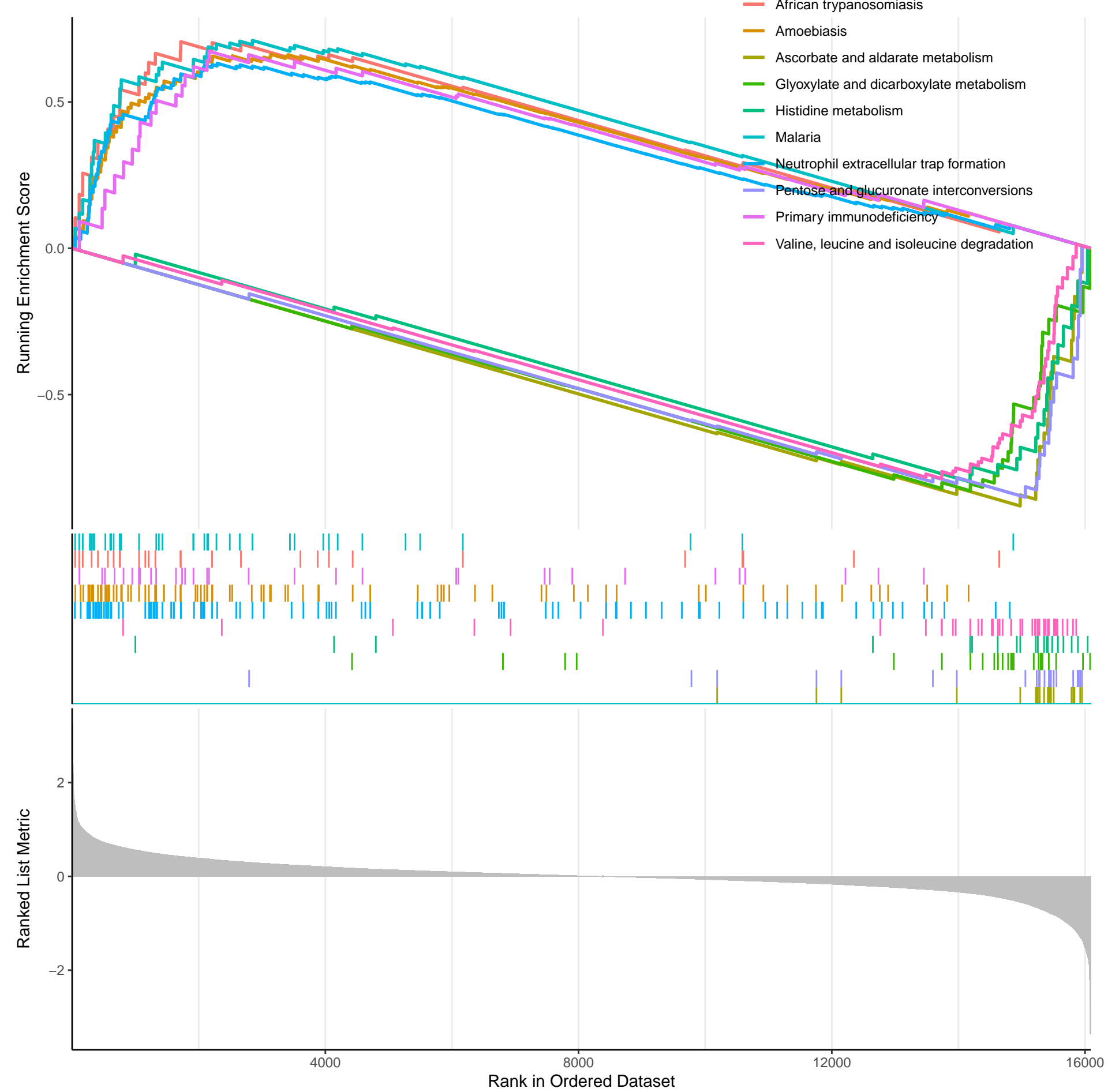

Supplement: Supplementary file 1 [file DataSheet3.ZIP › Figure1 and 10data and R code/Figure10 data and Rcode/2.MTOR_all_GSEA.pdf]

Running Enrichment Score

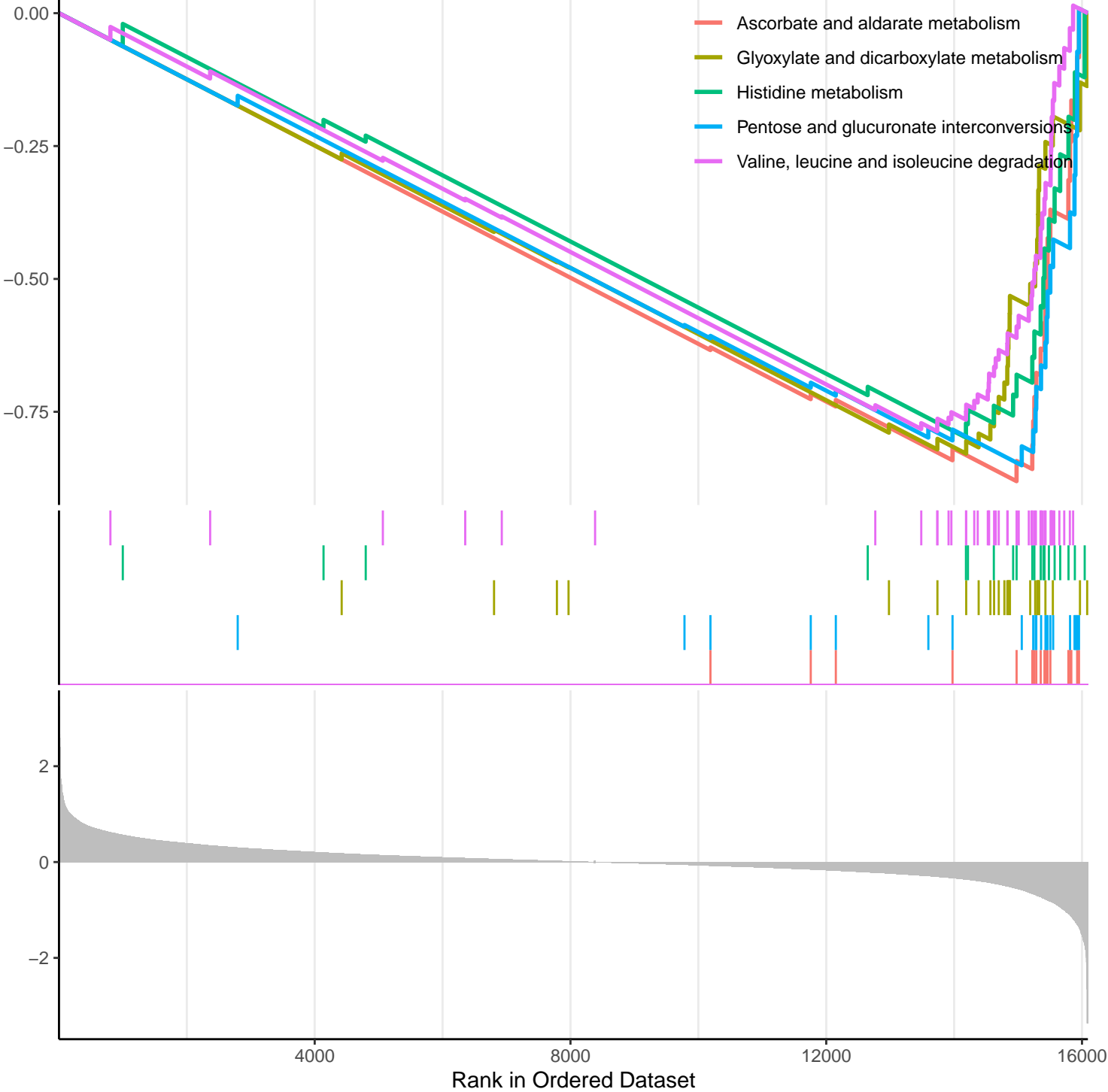

Supplement: Supplementary file 1 [file DataSheet3.ZIP › Figure1 and 10data and R code/Figure10 data and Rcode/2.MTOR_down_GSEA.pdf]

Running Enrichment Score

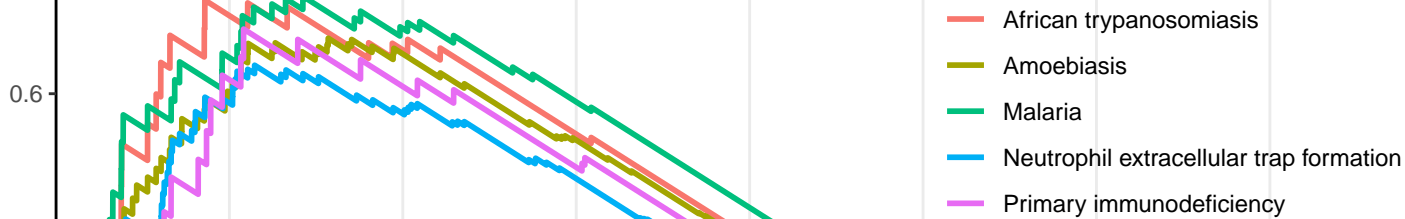

Ranked List Metric

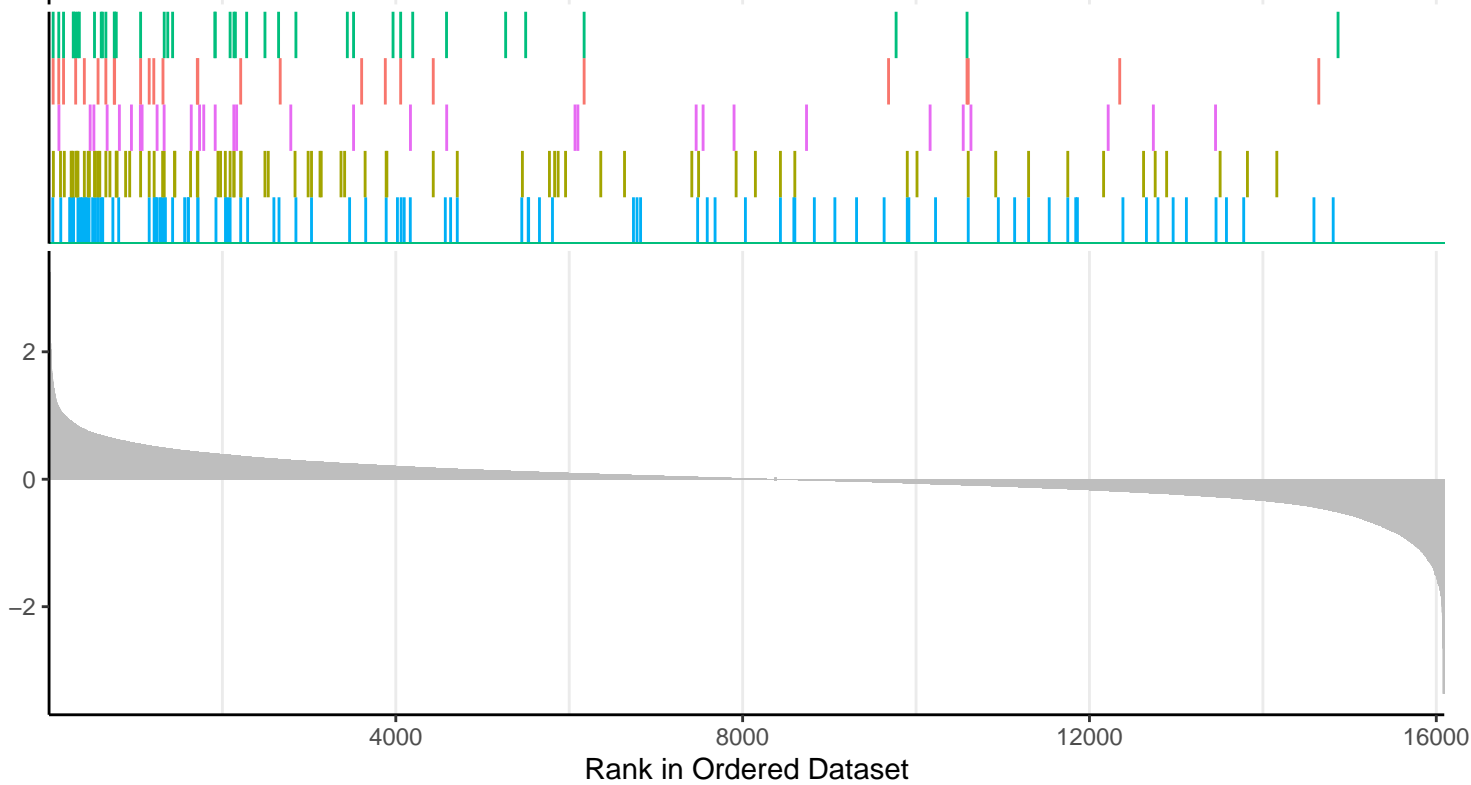

Supplement: Supplementary file 1 [file DataSheet3.ZIP › Figure1 and 10data and R code/Figure10 data and Rcode/2.MTOR_up_GSEA.pdf]

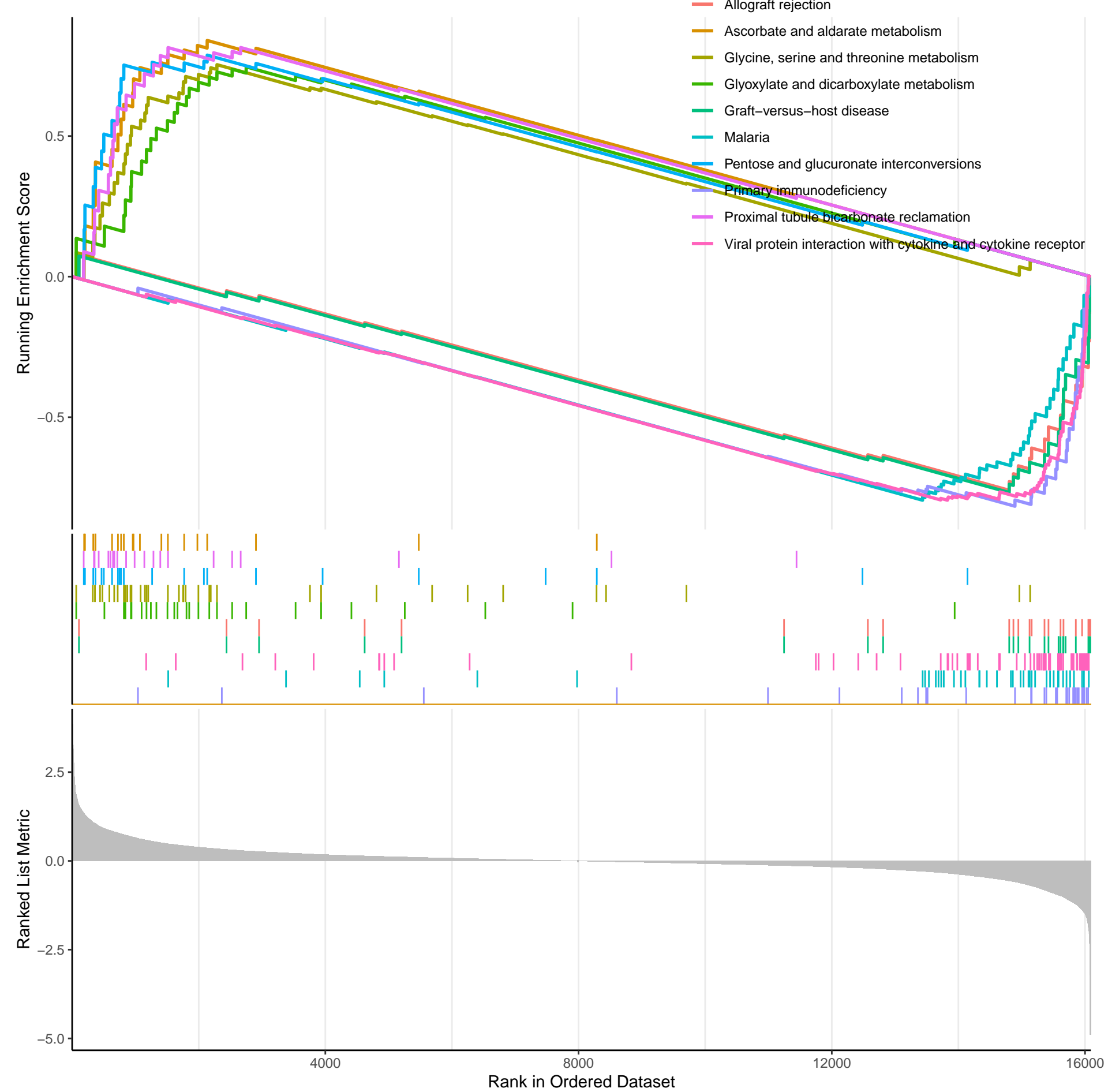

Supplement: Supplementary file 1 [file DataSheet3.ZIP › Figure1 and 10data and R code/Figure10 data and Rcode/2.VDR_all_GSEA.pdf]

Running Enrichment Score

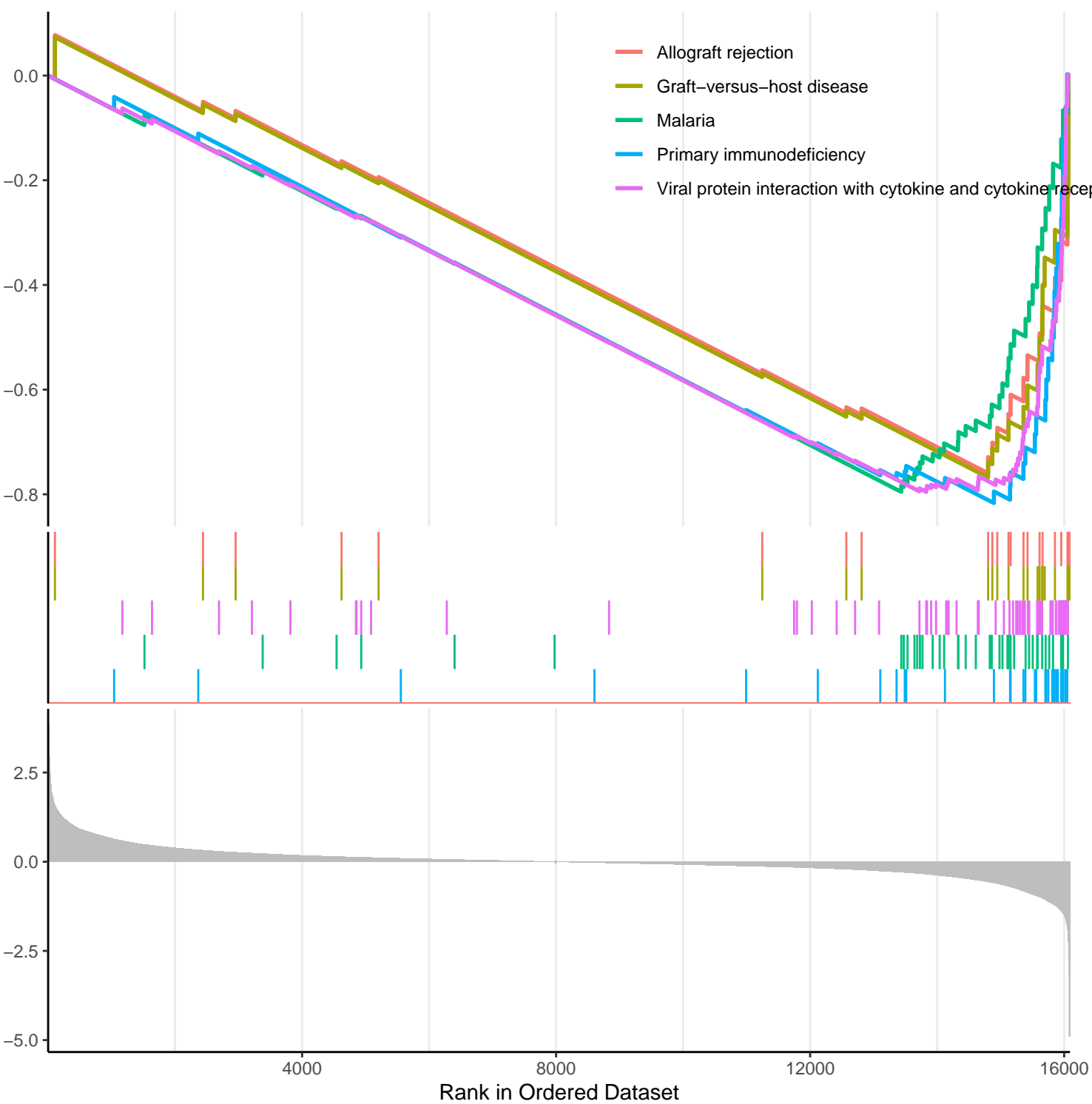

Supplement: Supplementary file 1 [file DataSheet3.ZIP › Figure1 and 10data and R code/Figure10 data and Rcode/2.VDR_down_GSEA.pdf]

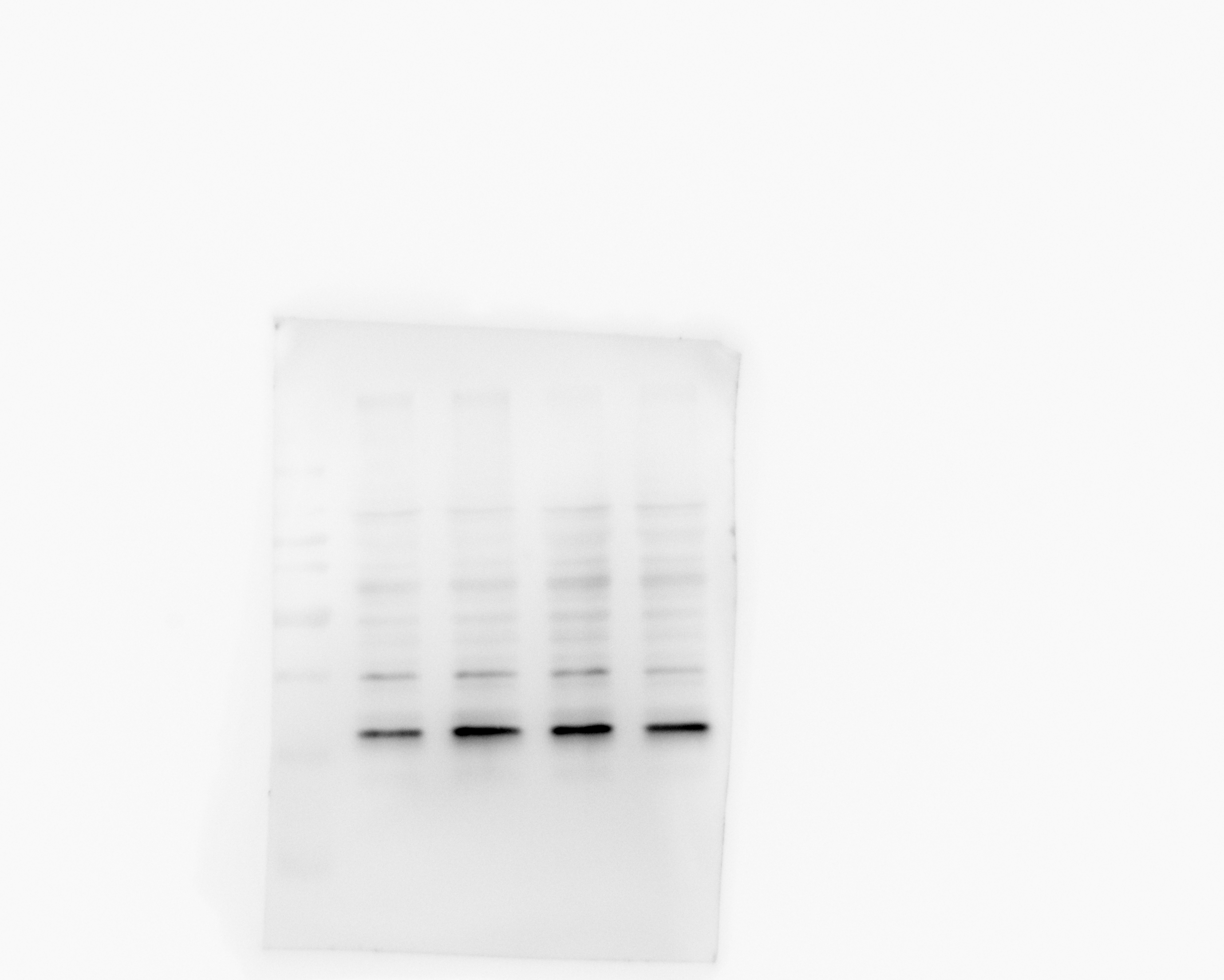

Supplement: Supplementary file 2 [file DataSheet4.ZIP › Fig8complete blot/MPC5-4EBP1-1.tif]

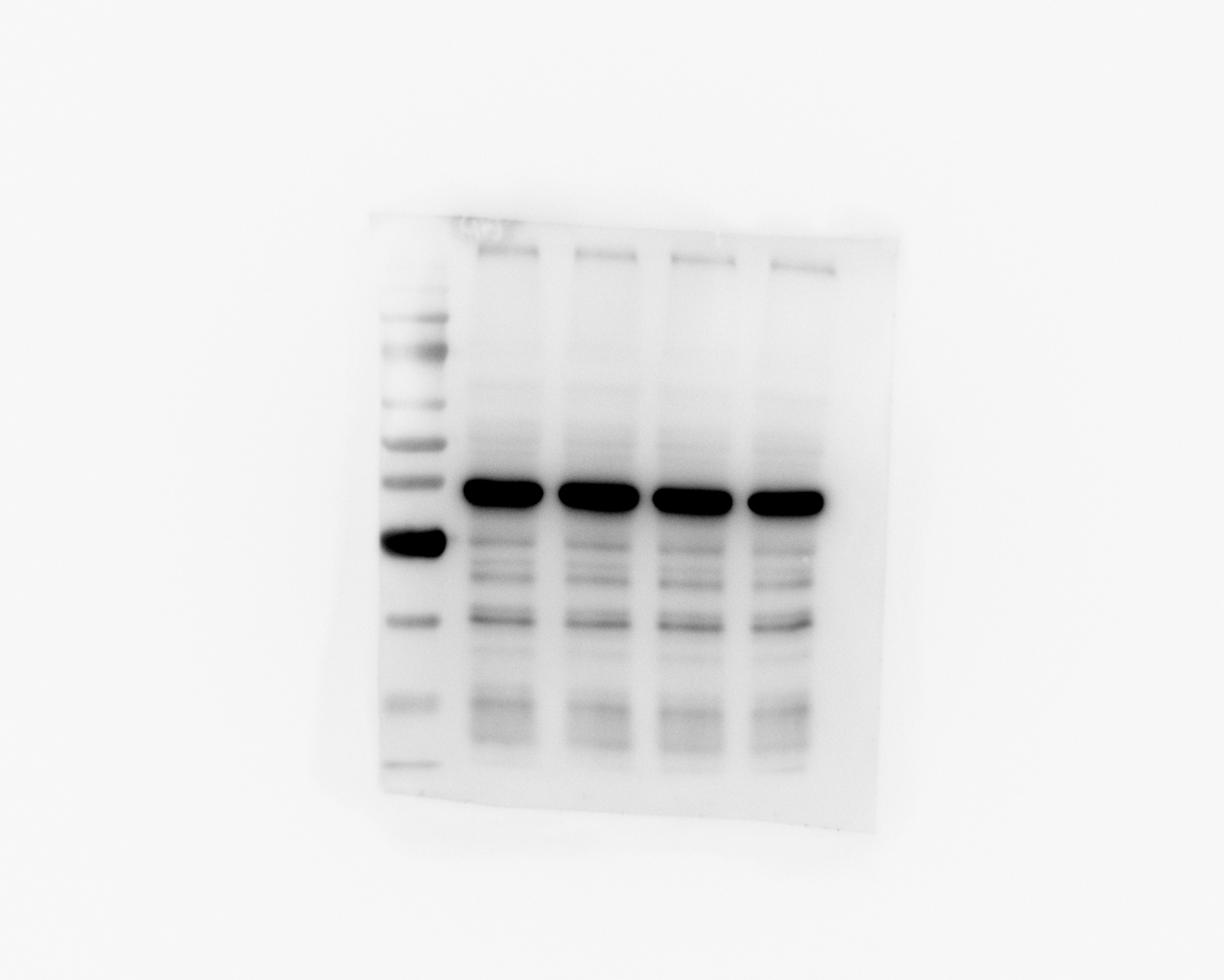

Supplement: Supplementary file 2 [file DataSheet4.ZIP › Fig8complete blot/MPC5-GAPDH-1.tif]

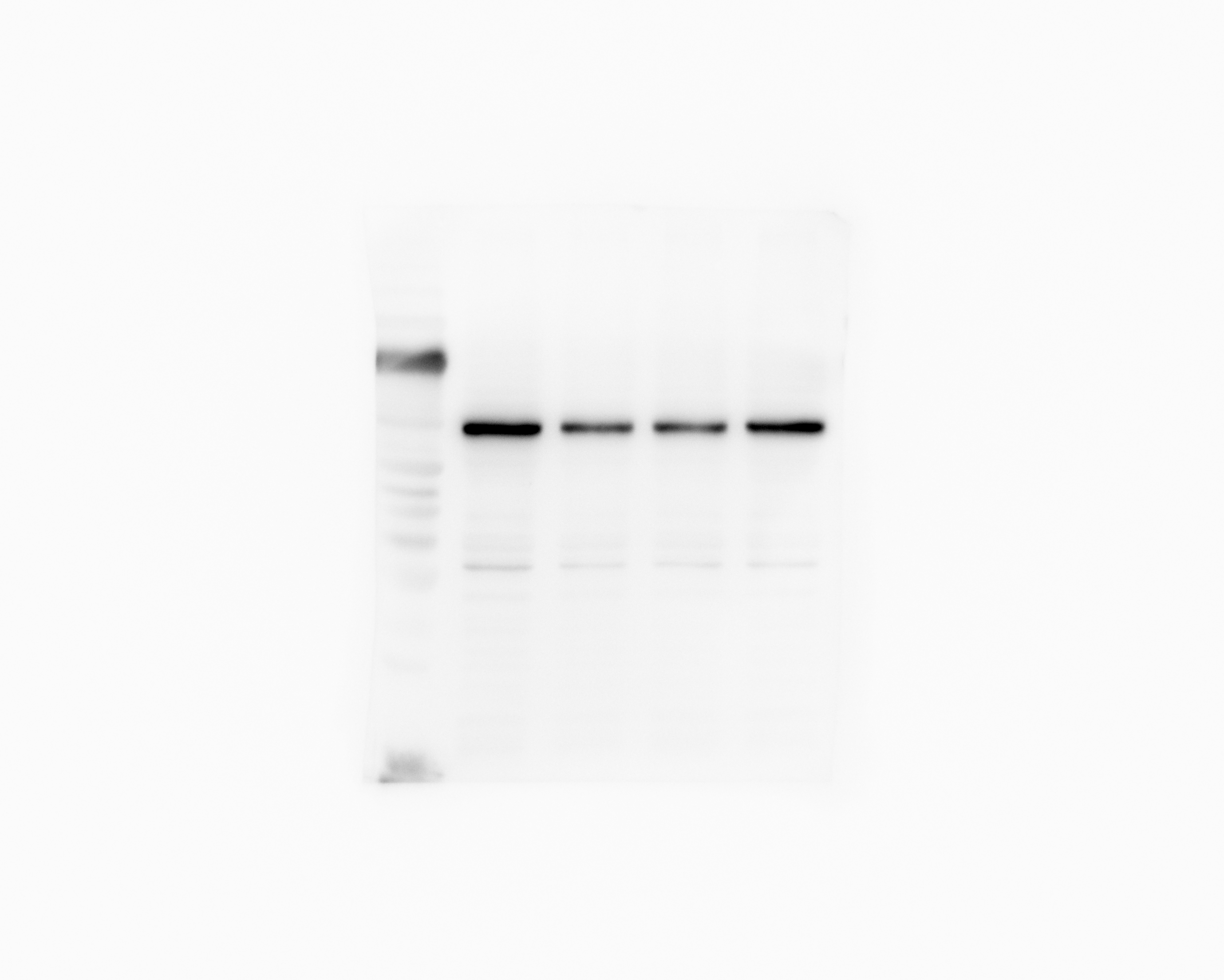

Supplement: Supplementary file 2 [file DataSheet4.ZIP › Fig8complete blot/MPC5-VDR-1.tif]

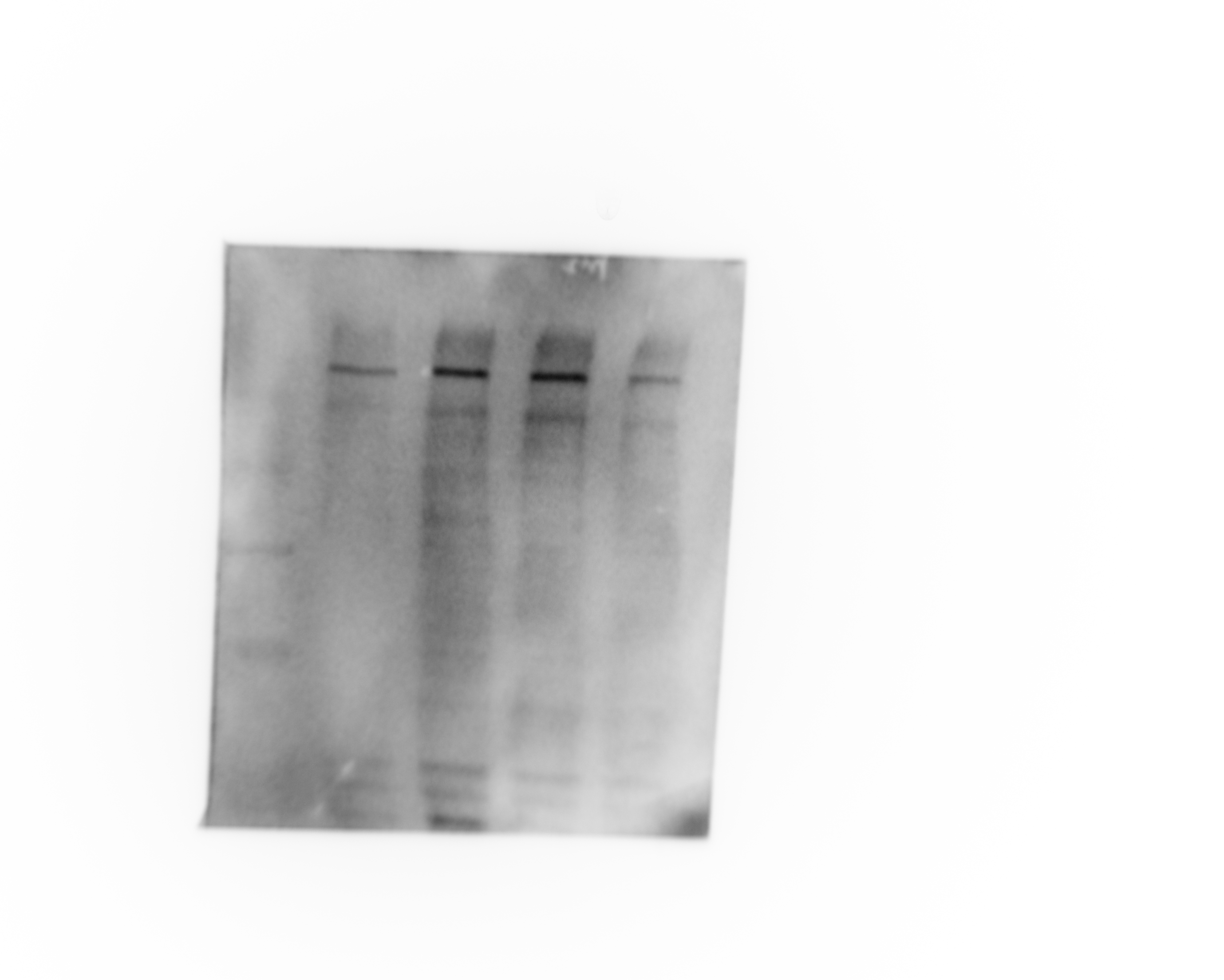

Supplement: Supplementary file 2 [file DataSheet4.ZIP › Fig8complete blot/MPC5-mTOR-1.tif]

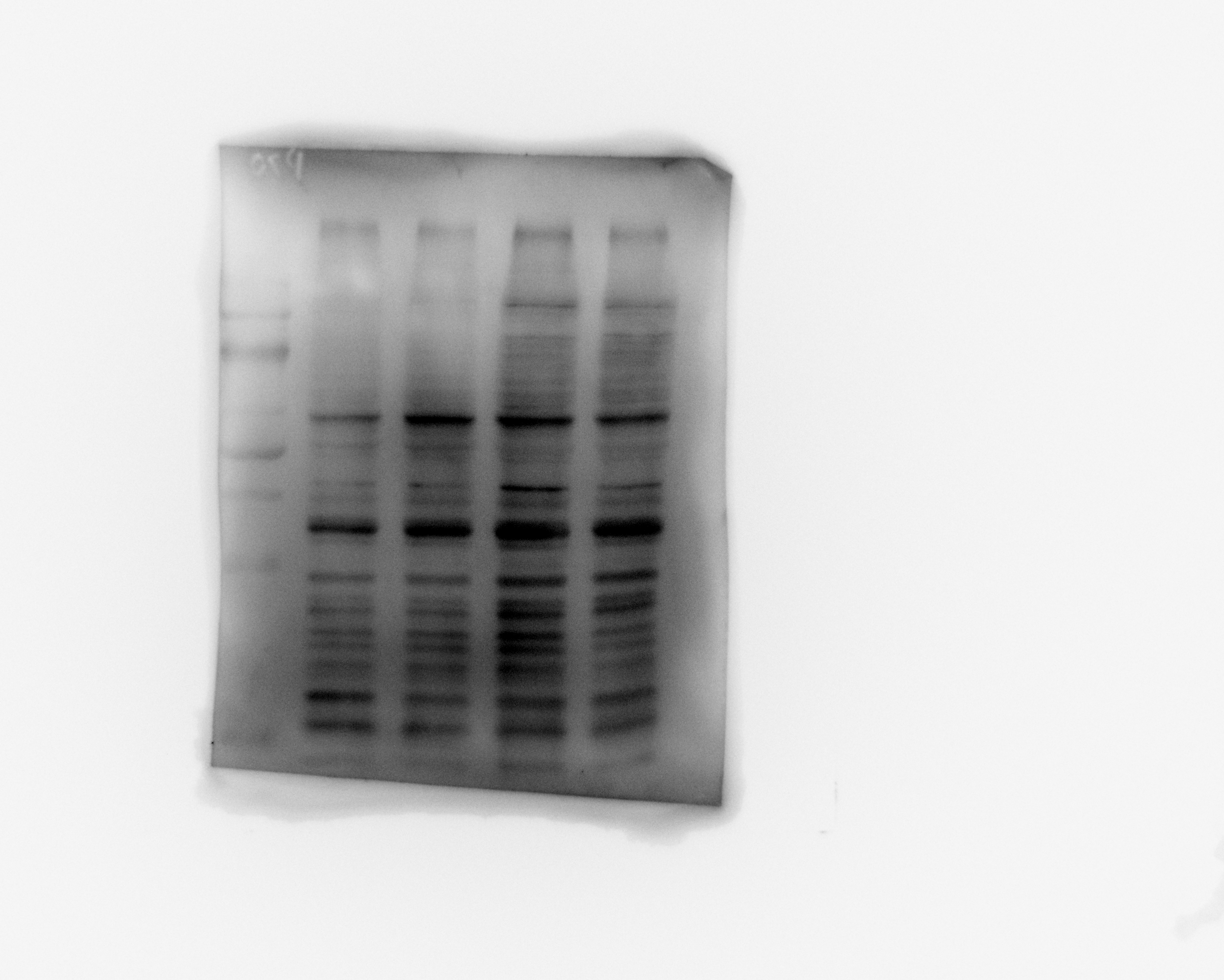

Supplement: Supplementary file 2 [file DataSheet4.ZIP › Fig8complete blot/MPC5-p70s6k-1.tif]

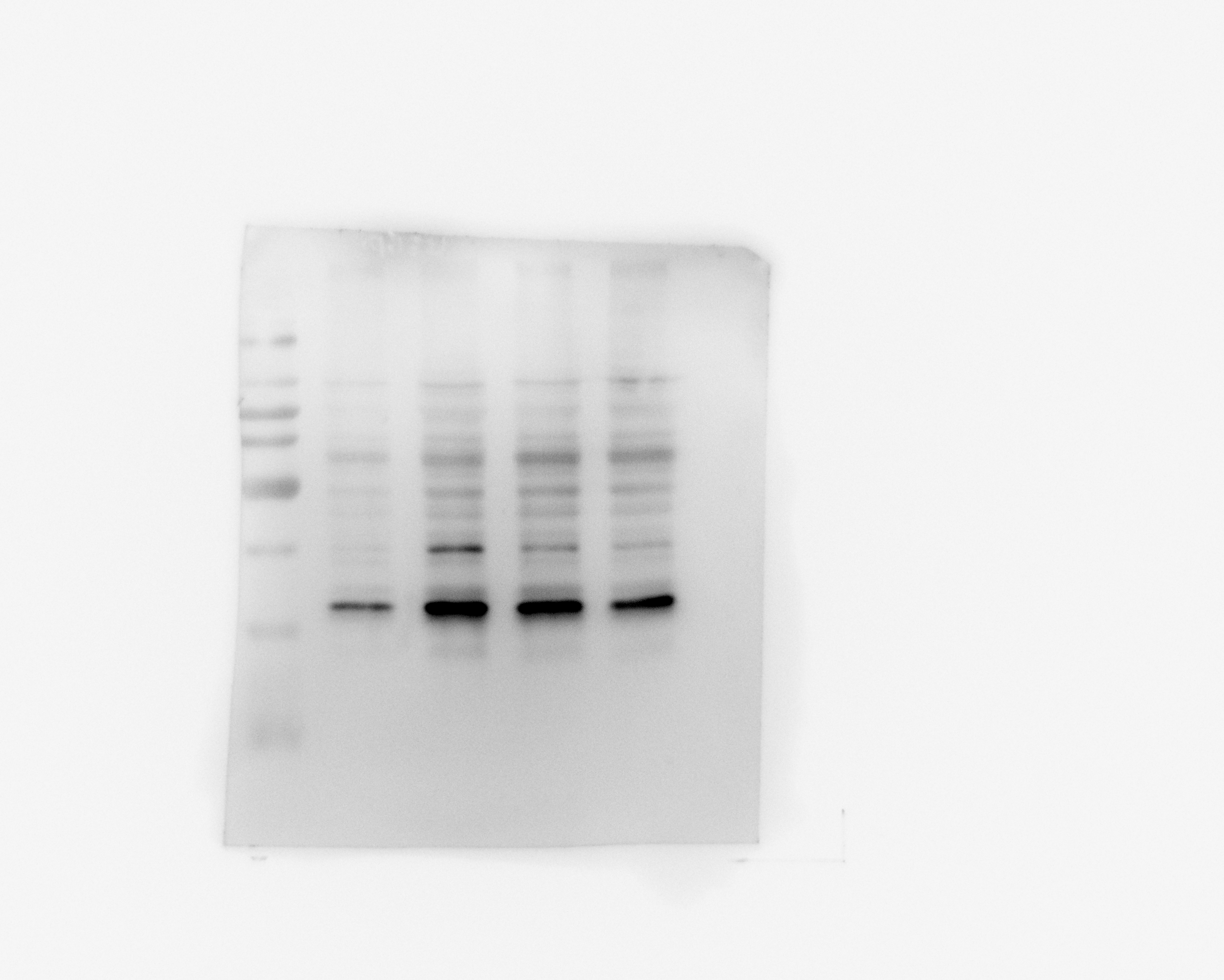

Supplement: Supplementary file 2 [file DataSheet4.ZIP › Fig8complete blot/SV40-4EBP1-2.tif]

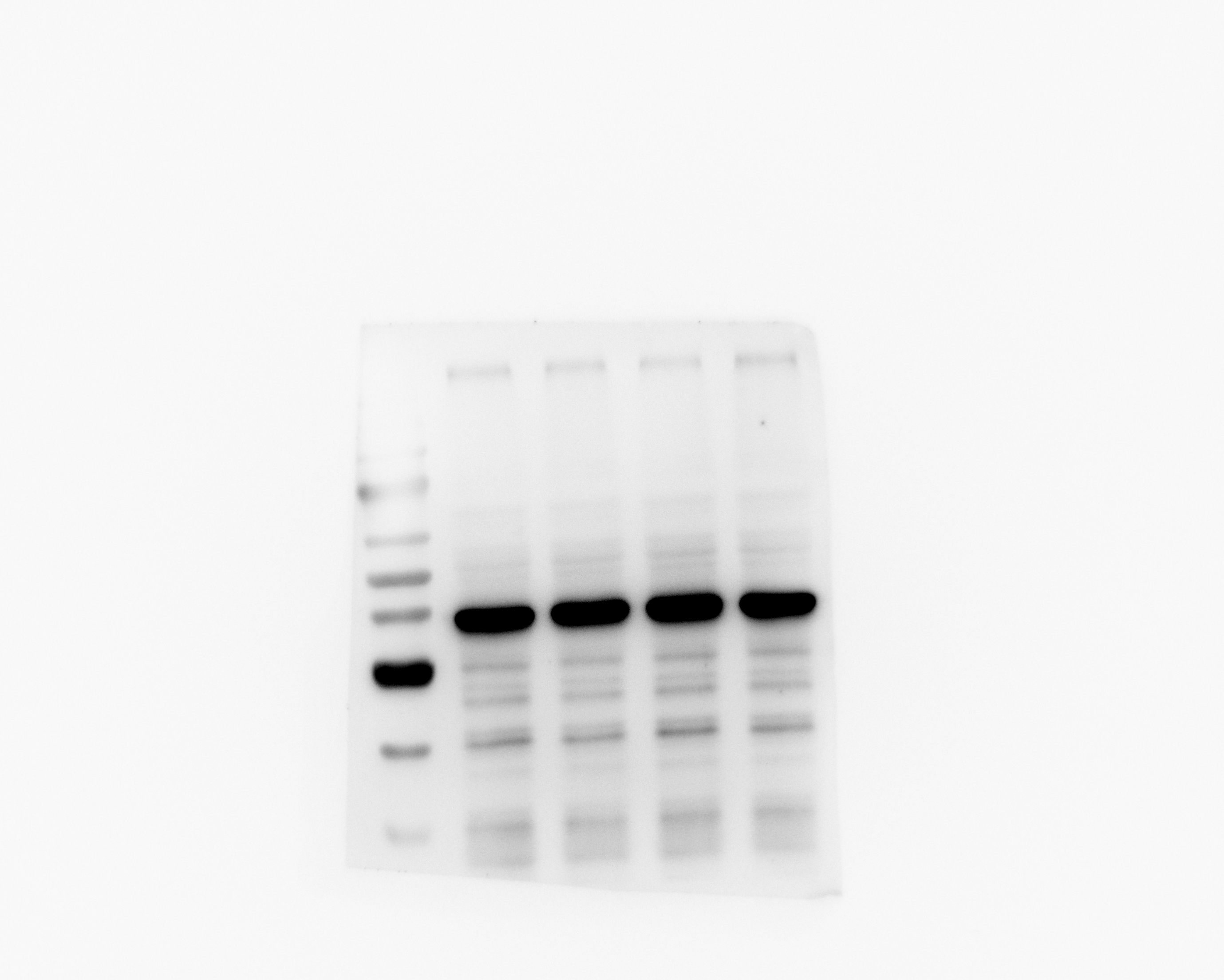

Supplement: Supplementary file 2 [file DataSheet4.ZIP › Fig8complete blot/SV40-GAPDH-2.tif]

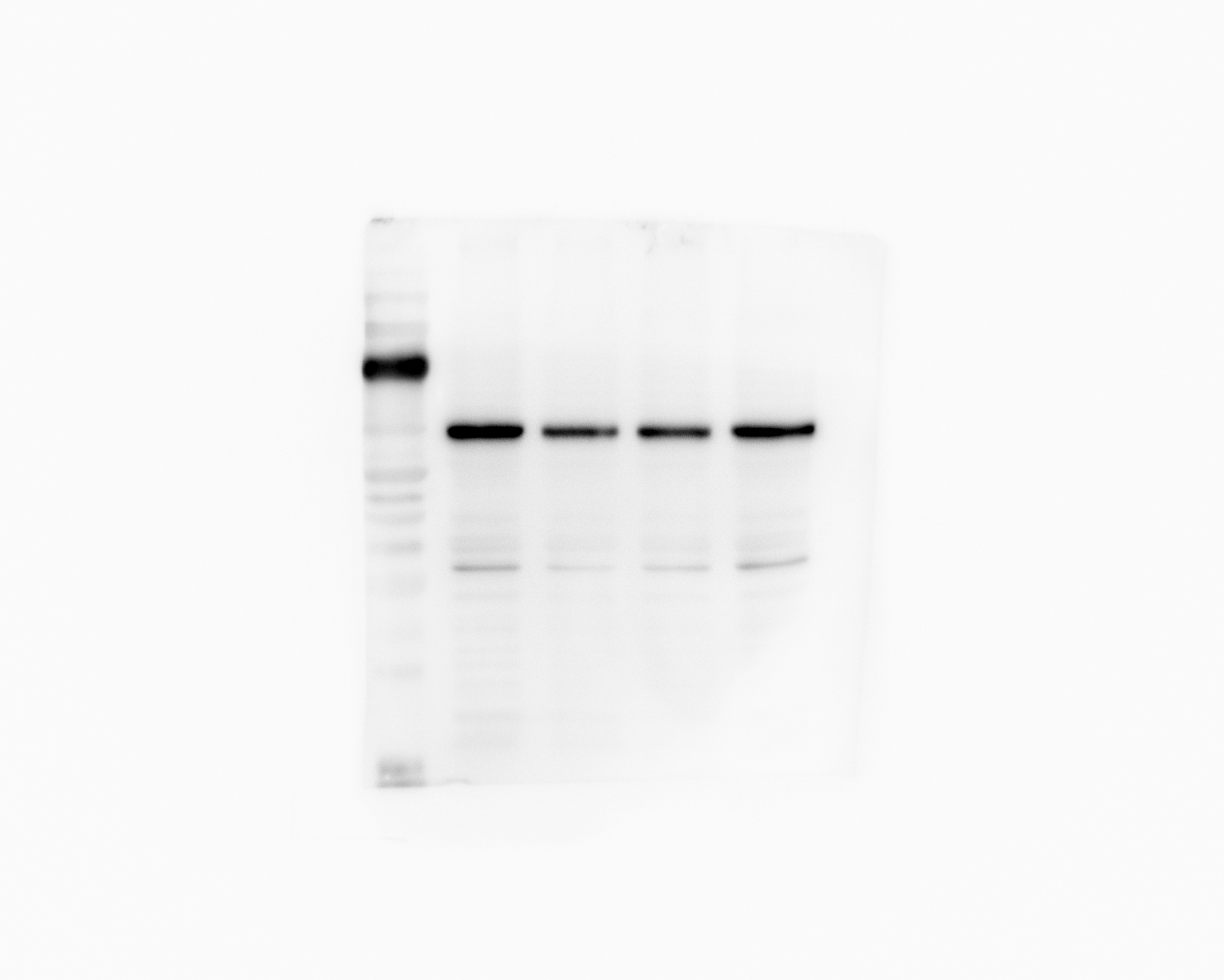

Supplement: Supplementary file 2 [file DataSheet4.ZIP › Fig8complete blot/SV40-VDR-2.tif]

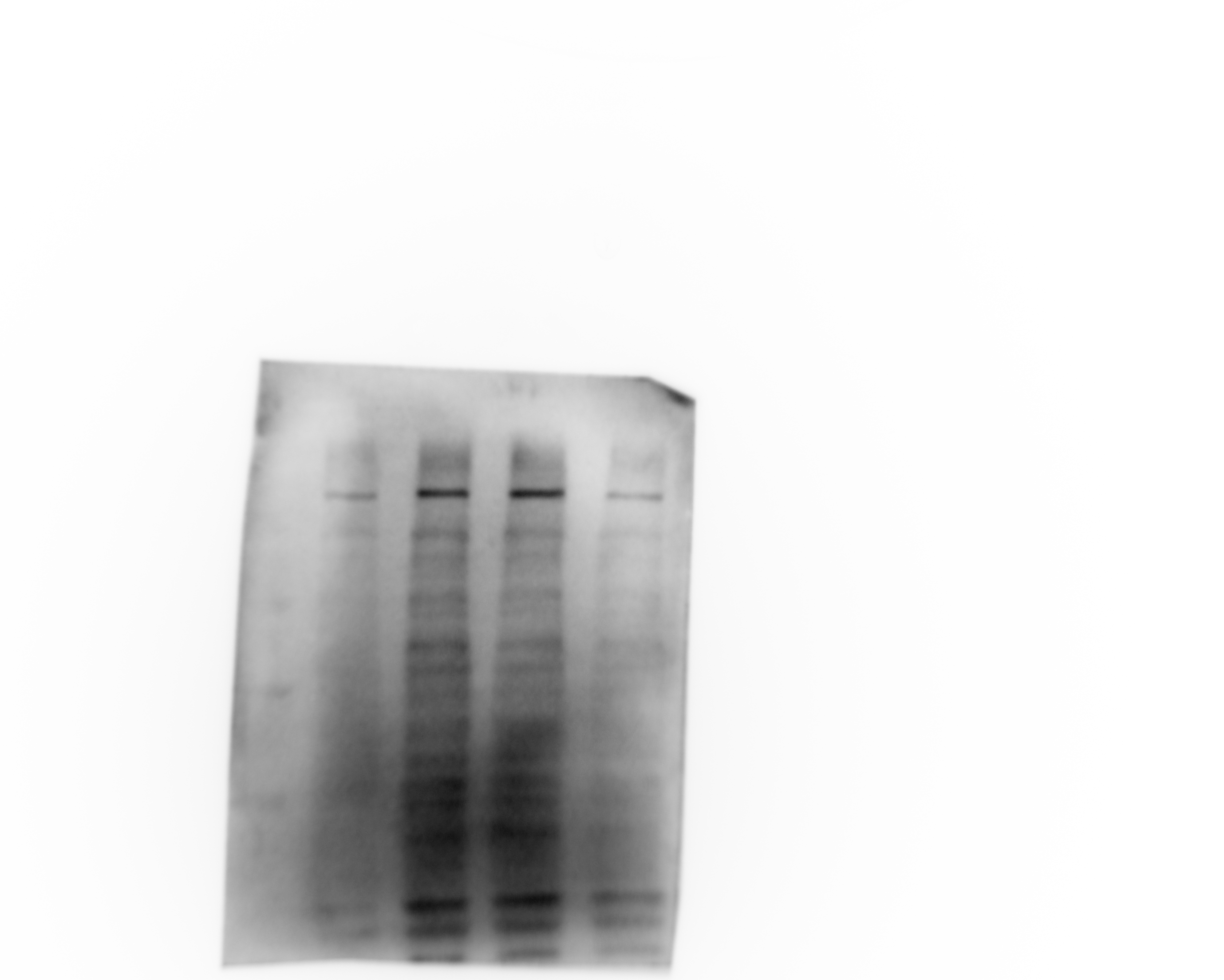

Supplement: Supplementary file 2 [file DataSheet4.ZIP › Fig8complete blot/SV40-mTOR-2.tif]

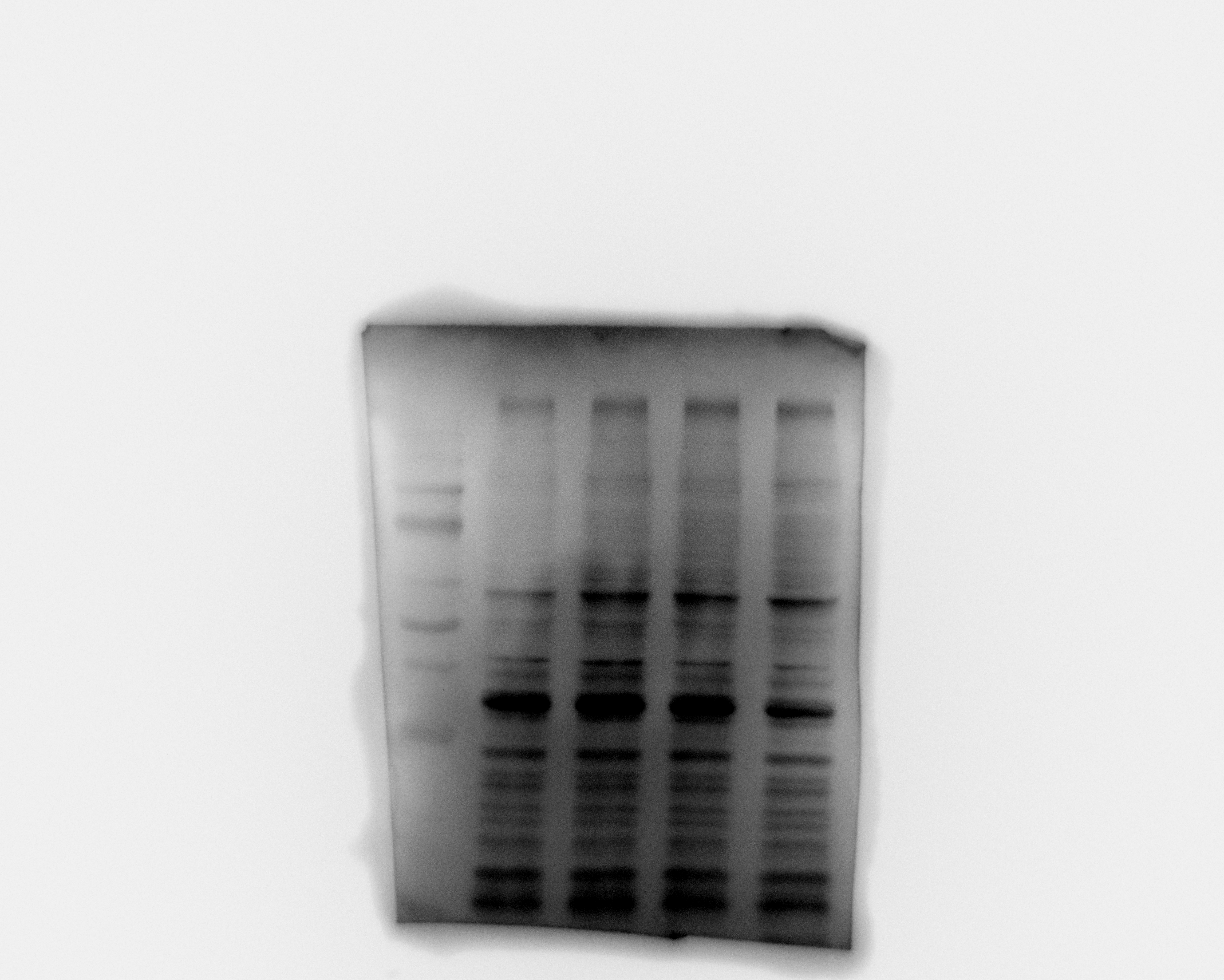

Supplement: Supplementary file 2 [file DataSheet4.ZIP › Fig8complete blot/SV40-p70s6k-2.tif]

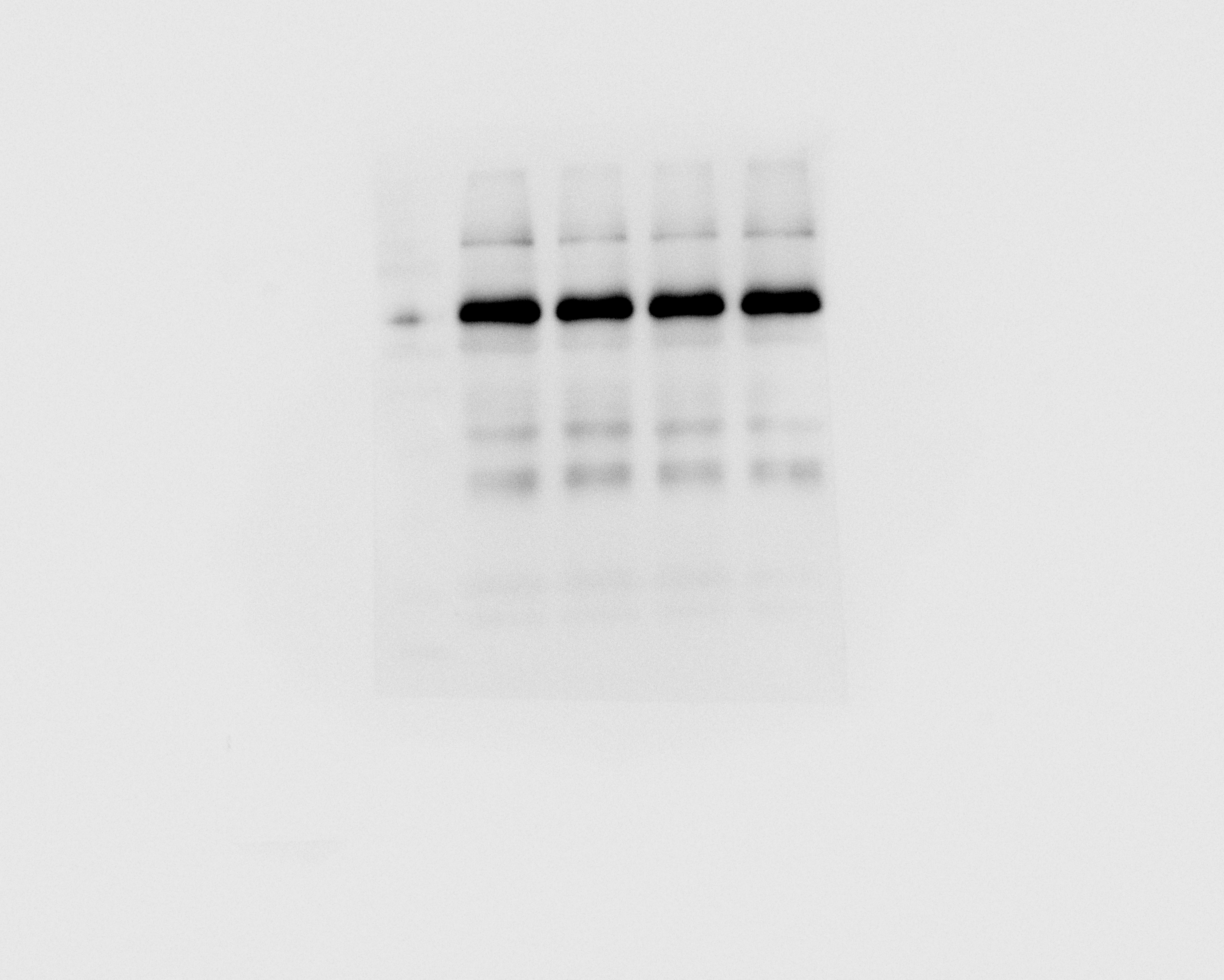

Supplement: Supplementary file 3 [file DataSheet5.ZIP › Fig9complete blot/MPC5-GAPDH-3.tif]

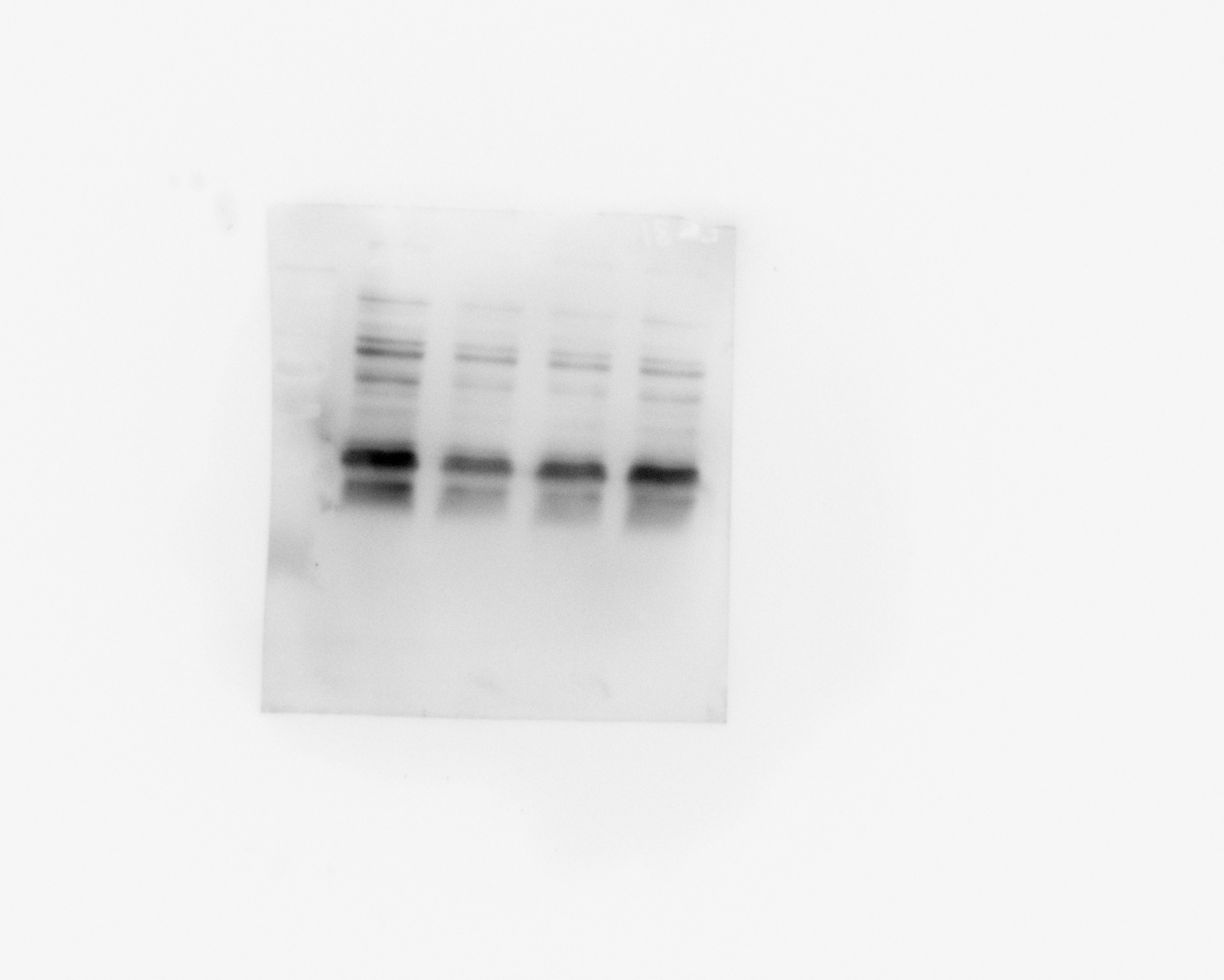

Supplement: Supplementary file 3 [file DataSheet5.ZIP › Fig9complete blot/MPC5-LC3I-3.tif]

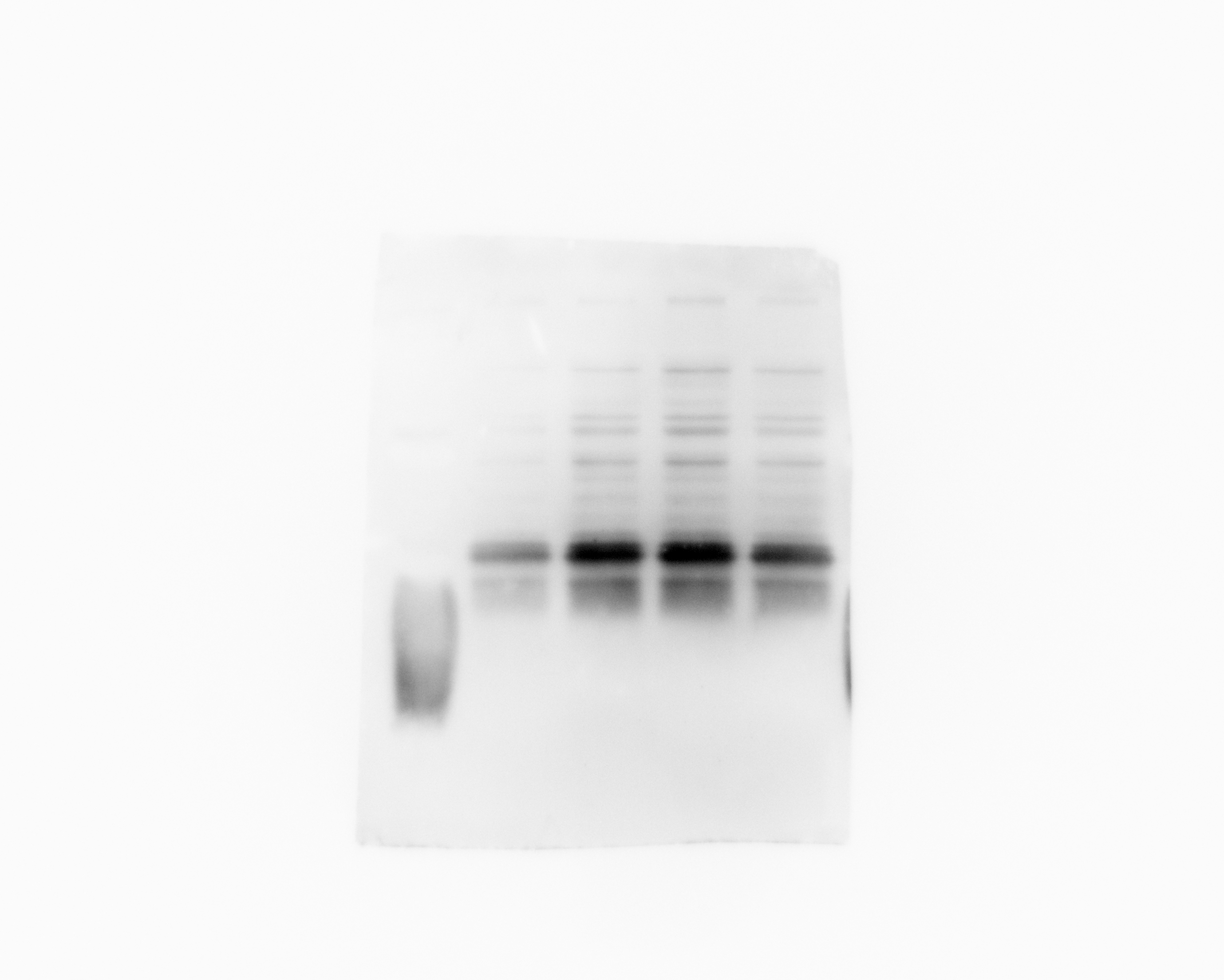

Supplement: Supplementary file 3 [file DataSheet5.ZIP › Fig9complete blot/MPC5-LC3II-3.tif]

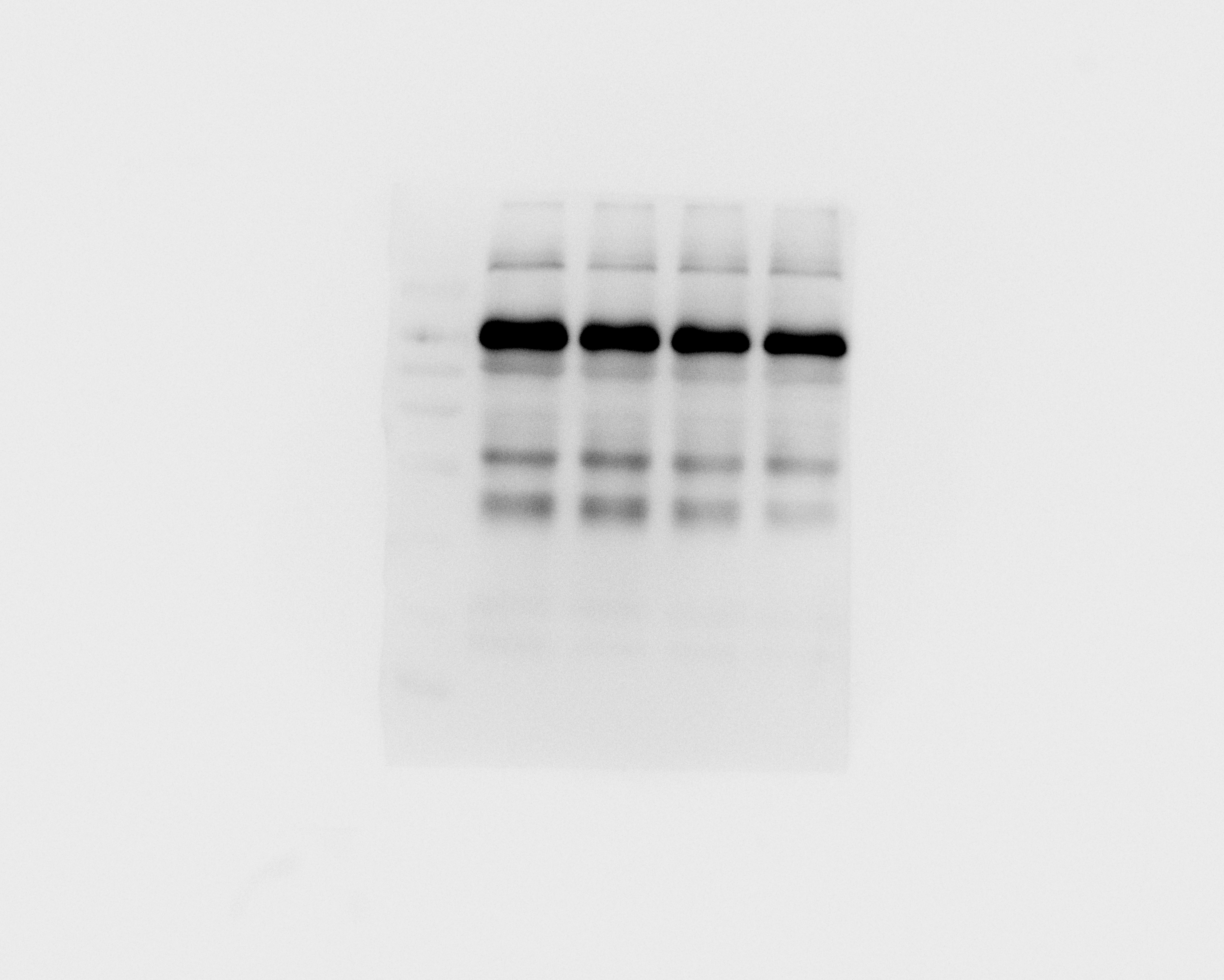

Supplement: Supplementary file 3 [file DataSheet5.ZIP › Fig9complete blot/SV40-GAPDH-3.tif]

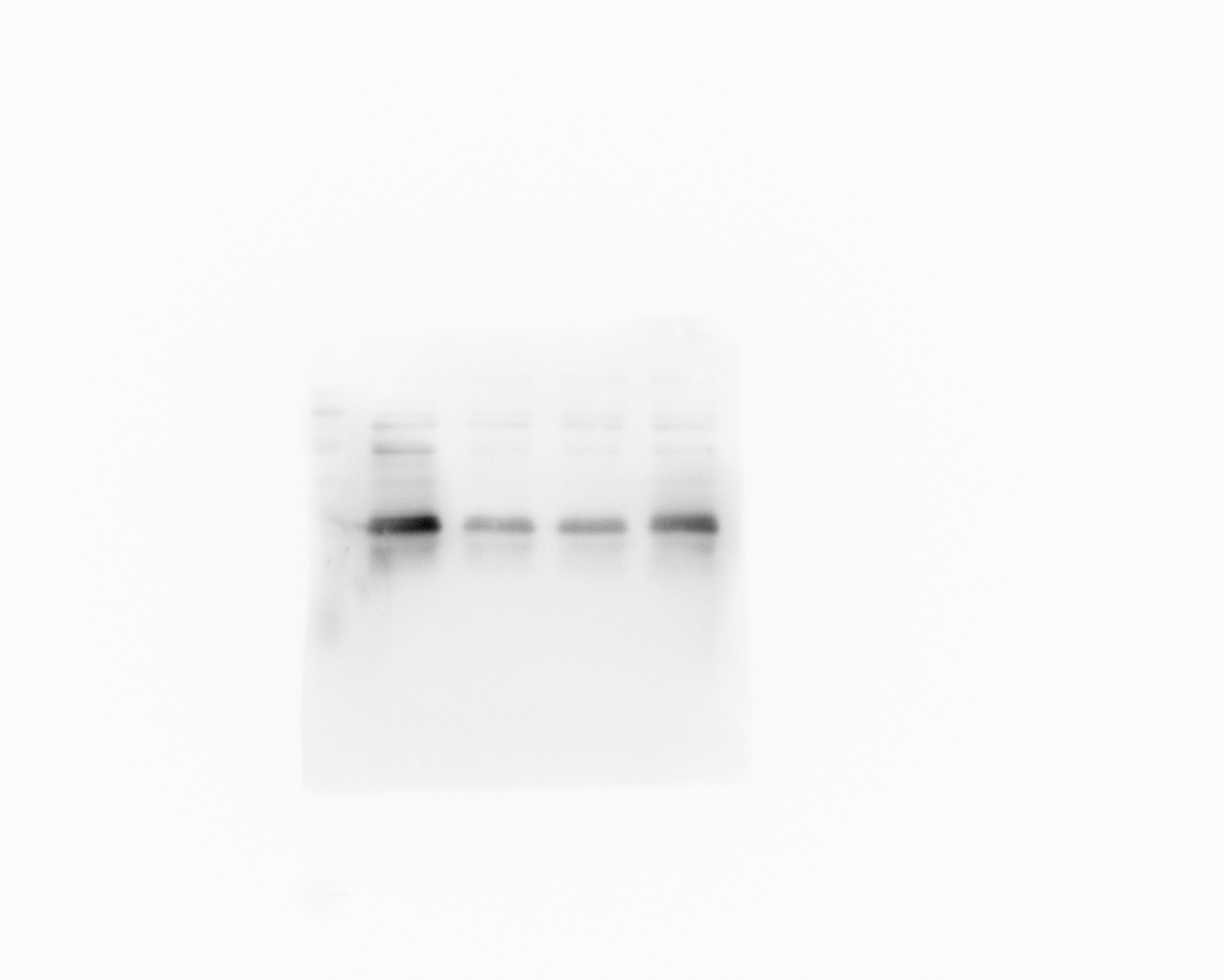

Supplement: Supplementary file 3 [file DataSheet5.ZIP › Fig9complete blot/SV40-LC3I-3.tif]

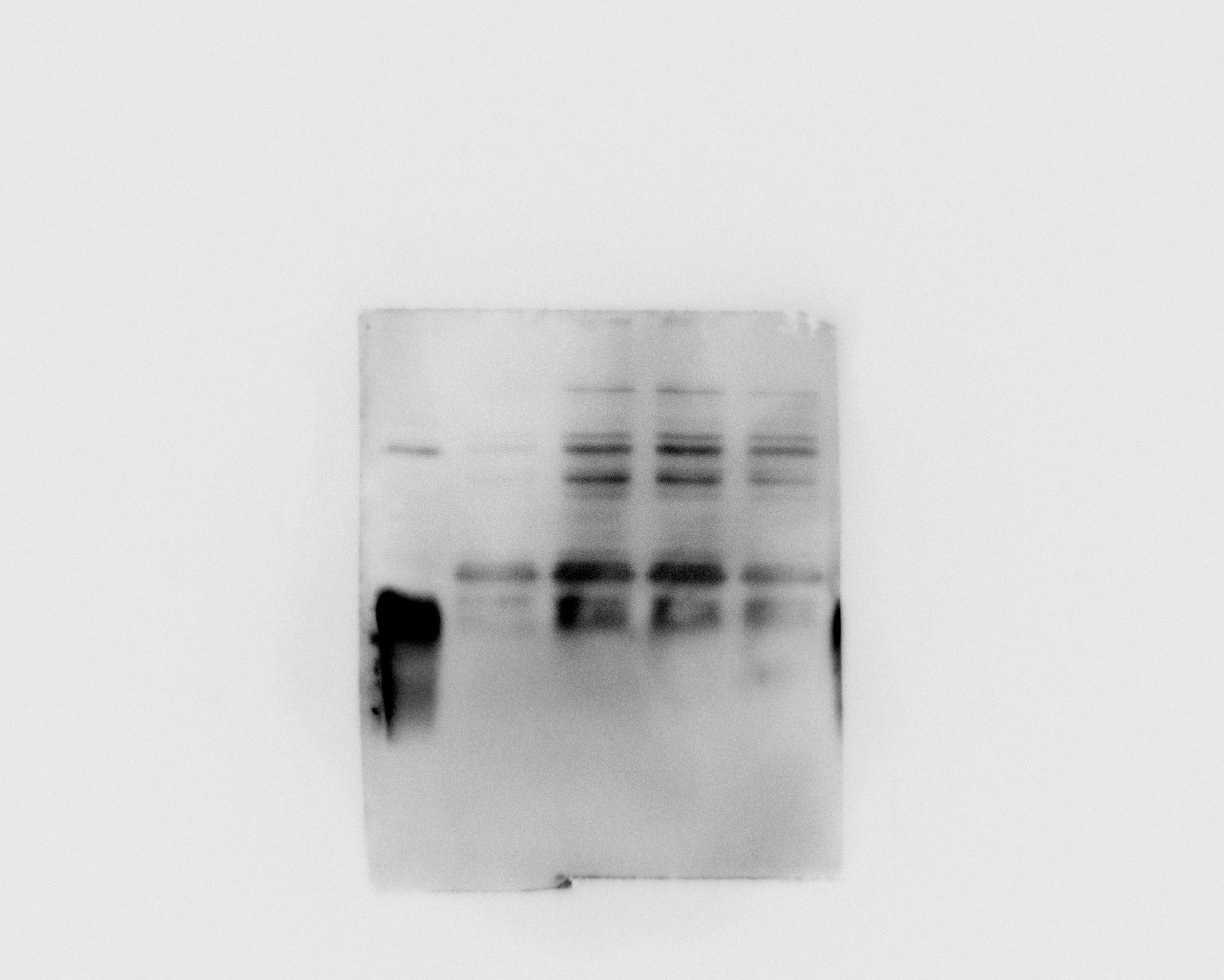

Supplement: Supplementary file 3 [file DataSheet5.ZIP › Fig9complete blot/SV40-LC3II-3.tif]
