# Supplementary material for: Unraveling DDIT4 in the VDR-mTOR pathway: a novel target for drug discovery in diabetic kidney disease
Source: Front Pharmacol. 2024 Mar 19;15:1344113. doi: 10.3389/fphar.2024.1344113 (PMC10985261; doi:10.3389/fphar.2024.1344113)
Supplement: Supplementary file 4 [file DataSheet1.DOCX]

Supporting Information

1. *Experimental materials*

The clinical samples were from patients who were admitted to the hospital and diagnosed as diabetes nephropathy by renal biopsy (according to the relevant standards in the 2021 version of the Chinese diabetes nephropathy clinical diagnosis and treatment guidelines). The tissue of patients with minimal change disease (MCD) served as a control.

The experimental animals were purchased from Beijing Charles River Laboratories (CRL) Experimental Animal Technology Co., Ltd., including 20-week-old male SPF db/db mice and their littermates with the m/m genotype. Each week, the weight and eating habits, mental state, fur color, and activity level of the mouse groups were measured. Starting from the 20th week, urine samples were collected using metabolic cages over a 12-hour period, during which the mice had access to water but were deprived of food. After a 12-hour fasting period, fasting blood glucose (FBG) levels were measured. At the end of the experiment, serum and kidney tissue samples were collected. The Beckman AU5800 fully automated biochemical analyzer was used to measure blood glucose levels in mice. The Mindray fully automated biochemical analyzer was used to measure urinary microalbumin (mAlb). Serum creatinine in mice was measured using the creatinine assay kit and the muscle amino oxidase method, without the need for serum sample dilution, following the instructions provided with the kit. A 1% concentration of FITC-insulin solution was injected into the posterior eye of the mice, while they were in an awake state, using a fructose solution (3.74μl/g of mouse weight), followed by immediate timing. Samples of approximately 25μL were collected at 11 time points: 1 min, 2 min, 3 min, 4 min, 5 min, 7 min, 10 min, 15 min, 35 min, 55 min, and 75 min, and placed in anticoagulant centrifuge tubes. The tubes were then centrifuged at 3000 rpm for 5 min, and 2 μL of plasma sample + 38μL of HEPES solution were taken from each time point and placed in a 96-well plate. The control group consisted of mixing 1 μL of 1% fructose stock solution with 199μL of HEPES solution (1:200) at the corresponding time points, and 40μL of the mixture was aspirated into the control wells of three 96-well plates. The fluorescence values of each time point in the 96-well plates were measured using the Tecan Spark multifunctional microplate reader. Finally, the plasma fluorescence data was fitted to a two-phase exponential decay curve using GraphPad Prism 8.0 software to calculate the glomerular filtration rate (GFR)[1].

The mouse immortalized kidney podocyte line MPC5 cells were purchased from ATCC, and the mouse glomerular mesangial cell line SV40-MES-13 cells were purchased from Shanghai Fuheng Cell Bank.

DMEM high sugar medium and fetal bovine serum (FBS) were purchased from Hyclone Company in the United States, and DMEM/F12 medium was purchased from Shanghai Enzymatic Biotechnology Co., Ltd. 100 × Penicillin streptomycin solution was purchased from Shanghai Sangong Biotechnology Co., Ltd. 0.25% trypsin and protein loading buffer were purchased from Beyotime Biotechnology Co., Ltd. Glucose and mannitol were purchased from MedChemexpress (MCE) Biotechnology Co., Ltd. Lipofectamine 2000 and Trizol reagents were purchased from Invitrogen. BCA protein quantitative kit and BSA reagent were purchased from Biosharp Co., Ltd. 30% acrylamide and 10% SDS were purchased from Shanghai Sinopharm Reagent Co., Ltd. 1.5 M Tris HCl and 1.0 M Tris HCl were purchased from Beijing Solebar Technology Co., Ltd. 10% ammonium persulfate and TEMED were purchased from SIGMA. Antibody for mTOR was procured from Abcam (Cambridge, MA, UK), and antibodies for VDR, p70s6k, LC3I and LC3II were from Affinity Biosciences (Cincinnati, OH, USA), and antibodies for 4EBP1 and GAPDH were from Proteintech (Rosemont, IL, USA). ECL luminous solution was purchased from Beijing Dingguo Changsheng Biotechnology Co., Ltd. SYBR Green PCR kit and reverse transcription kit were purchased from Thermo Fisher Scientific.

1. *Supplementary Table 1: qRT-PCR Primer Sequences*

| **Gene** | **Forward Primer** | **Reverse Primer** |
| --- | --- | --- |
| DDIT4 | GTCCCTGCCCGACTTTGA | CTCTTGCCTTGCTCCACA |
| VDR | ACCGCCTATCCAACACAC | ATCTCATTGCCGAACACC |
| mTOR | CAATGAGAGGAGGTGG | ACGGAGAACGAGGACAGC |
| p70s6k | TACAGAGACCTGAAGCCG | CTCCCAAAACTCCACCAAT |
| 4E-BP1 | CGGGAGGAACCAGGATTA | TTGGGGGACATAGAAGCA |
| GAPDH | GGTGAAGGTCGGTGTGGAACG | CTCGCTCCTGGAAGATGGTG |

**References**

[1] Z. Qi, I. Whitt, A. Mehta, J. Jin, M. Zhao, R.C. Harris, et al., Serial determination of glomerular filtration rate in conscious mice using FITC-inulin clearance, Am. J. Physiol. Renal Physiol. 286 (3) (2004) F590-596, https://doi.org/10.1152/ajprenal.00324.2003.
